# Redox Phenotyping of the Donor Heart: A New Paradigm for Precision Heart Transplantation

**DOI:** 10.3390/antiox15101261

**Published:** 2026-10-01

**Authors:** Tomasz Urbanowicz, Mariusz Kowalewski, Giuseppe Maria Raffa, Beata Krasińska, Calogera Pisano, Vincenzo Nuzzi, Mansur Rahnama, Piotr Suwalski, Zbigniew Krasiński, Ewelina Grywalska

**Affiliations:** 1 Cardiac Surgery and Transplantology Department, Poznan University of Medical Sciences, ½ Długa, 61-848 Poznań, Poland; 2 Thoracic Research Center, Collegium Medicum, Nicolaus Copernicus University, 85-067 Bydgoszcz, Poland; 3 Department of Cardiac Surgery, Center of Postgraduate Medical Education, Central Clinical Hospital of the Ministry of Interior, 02-507 Warszawa, Poland; 4 Department of Research, IRCCS ISMETT, 90127 Palermo, Italy; 5 Department of Precision Medicine in Medical Surgical and Critical Area (Me.Pre.C.C.), University of Palermo, 90134 Palermo, Italy; 6 Department of Hypertensiology, Angiology, and Internal Medicine, Poznan University of Medical Sciences, ½ Dluga Street, 61-848 Poznań, Poland; 7 Clinical Cardiology and Heart Failure Unit, IRCCS ISMETT, 90127 Palermo, Italy; 8 Department of Dental Surgery, Medical University of Lublin, 6 Chodźki Street, 20-093 Lublin, Poland; 9 Department of Vascular, Endovascular Surgery, Angiology and Phlebology, Poznan University of Medical Science, ½ Dluga Street, 61-848 Poznan, Poland; 10 Department of Experimental Immunology, Faculty of Medical Sciences, Medical University of Lublin, 20-093 Lubin, Poland

**Keywords:** oxidative stress, ischemia–reperfusion injury, machine perfusion, mitochondrial dysfunction, endothelial dysfunction, extracellular vesicles, metabolomics, oxidative biomarkers, artificial intelligence, precision medicine

## Abstract

Heart transplantation remains the definitive treatment for selected patients with advanced heart failure, yet current donor heart assessment relies predominantly on clinical, functional, and biochemical variables that incompletely reflect the biological resilience of the graft. Increasing evidence indicates that oxidative stress represents a central mechanism linking donor characteristics, brain death, ischemia–reperfusion injury, endothelial dysfunction, mitochondrial impairment, inflammation, and regulated cell death. These interconnected processes collectively determine graft susceptibility to preservation injury and post-transplant dysfunction. This review introduces redox phenotyping as a novel conceptual framework for donor heart evaluation, integrating oxidative injury, antioxidant defenses, mitochondrial competence, endothelial integrity, metabolic adaptation, and redox-sensitive molecular signaling into a multidimensional assessment of graft biology. We summarize the mechanisms underlying donor heart oxidative remodeling throughout life, the molecular basis of ischemia–reperfusion injury, and emerging biomarkers, including metabolomics, lipidomics, extracellular vesicles, cell-free nucleic acids, and mitochondrial biomarkers. We further discuss how ex vivo machine perfusion enables real-time biological assessment and targeted therapeutic intervention, transforming organ preservation into a platform for dynamic redox characterization. Finally, we explore the integration of multi-omic profiling with artificial intelligence to enable individualized donor assessment and biological optimization.

## 1. Introduction

Heart transplantation remains the definitive treatment for carefully selected patients with advanced heart failure, providing substantial improvements in survival, functional capacity, and quality of life [1,2]. Despite remarkable progress in donor management, organ preservation, surgical techniques, and immunosuppressive therapy, primary graft dysfunction and chronic allograft injury continue to represent major barriers to further improving transplant outcomes [3,4]. Current donor heart evaluation is still based predominantly on demographic characteristics, conventional clinical variables, echocardiographic assessment, hemodynamic stability, and biochemical markers of myocardial injury [5,6]. Although these parameters identify overt structural or functional abnormalities, they provide only a limited representation of the donor heart’s biological condition and frequently fail to account for the considerable heterogeneity in post-transplant graft performance.

Increasing evidence indicates that the biological resilience of the donor heart is established long before organ procurement [7,8]. Throughout life, the myocardium is exposed to aging, cardiometabolic disease, environmental factors, systemic inflammation, and intermittent ischemia, progressively remodeling redox homeostasis [9,10]. These cumulative exposures shape mitochondrial function, antioxidant capacity, endothelial integrity, inflammatory responsiveness, and metabolic flexibility, thereby generating an individualized biological state that may critically influence the heart’s ability to tolerate brain death, ischemia, preservation, and reperfusion [11]. Consequently, donor hearts with apparently similar clinical characteristics may differ substantially in their intrinsic capacity to withstand transplantation-associated stress.

Brain death and organ procurement introduce an additional phase of profound oxidative challenge [12,13]. Massive catecholamine release, systemic inflammation, endothelial activation, mitochondrial dysfunction, and ischemia initiate a cascade of reactive oxygen species generation that is further amplified during preservation and reperfusion. Oxidative stress is therefore not merely a consequence of transplantation but a central biological process linking donor characteristics, preservation injury, innate immune activation, regulated cell death, and subsequent graft dysfunction [14,15]. The balance between oxidative injury and antioxidant defense ultimately determines graft resilience.

Recent advances in molecular profiling have created an unprecedented opportunity to directly characterize this biological state. High-resolution metabolomics, lipidomics, redox-sensitive biomarkers, extracellular vesicle analysis, cell-free nucleic acids, mitochondrial functional assessment, and single-cell technologies are beginning to reveal molecular signatures that cannot be captured by conventional clinical evaluation alone [16,17,18]. Simultaneously, ex vivo machine perfusion has transformed donor heart preservation from passive storage to a dynamic biological platform that enables continuous monitoring of metabolic activity, oxidative stress, and functional recovery [19].

These advances indicate that conventional donor assessment incompletely captures the graft’s biological quality. We propose the concept of redox phenotyping, defined as the integrated characterization of oxidative injury, antioxidant defenses, mitochondrial competence, metabolic adaptation, endothelial function, and redox-sensitive molecular signaling within the donor heart. Unlike isolated biomarkers, redox phenotyping considers oxidative biology as a multidimensional property that reflects both the cumulative biological history of the donor heart and its capacity to respond to transplantation-associated stress.

The objective of this review is to establish redox phenotyping as a unifying framework for contemporary donor heart assessment. We discuss the origins of oxidative remodeling in donor hearts, the molecular mechanisms underlying ischemia–reperfusion injury, emerging biomarkers in redox biology, and therapeutic strategies targeting oxidative stress during organ preservation. Finally, we explore how integration of molecular profiling, machine perfusion, and artificial intelligence may enable a transition from conventional donor selection toward biologically informed precision heart transplantation.

In practical terms, redox phenotyping should not be understood as a single biomarker measurement, but as a structured multidomain assessment of donor-heart biology. A clinically applicable phenotype could integrate markers of oxidative injury, antioxidant capacity, mitochondrial function, endothelial integrity, and metabolic adaptation obtained from donor blood, myocardial tissue, preservation fluid, or serial perfusate samples during ex vivo machine perfusion. These molecular data would be interpreted alongside conventional donor characteristics, ventricular function, hemodynamics, ischemic exposure, and functional responses during preservation. Importantly, the objective would not be to generate an additional binary criterion for organ acceptance, but to identify patterns of biological vulnerability and recovery that are not apparent from conventional assessment alone. Redox phenotyping should therefore be regarded as a testable framework whose individual components, optimal sampling strategy, analytical thresholds, and incremental prognostic value require prospective validation before clinical implementation.

### 1.1. Methodology

This narrative review was conducted to critically evaluate the emerging role of oxidative stress and redox biology in donor heart assessment and to develop a conceptual framework for redox phenotyping in precision heart transplantation. Rather than providing a comprehensive systematic review of all published studies, the objective was to integrate current mechanistic, translational, and clinical evidence into a biologically coherent model that links donor characteristics to graft resilience and post-transplant outcomes.

A structured literature search was performed using PubMed/MEDLINE as the primary database. Publications available through March 2026 were identified using combinations of the following keywords and Medical Subject Headings (MeSH): heart transplantation, donor heart, brain death, oxidative stress, reactive oxygen species, redox signaling, mitochondria, ischemia–reperfusion injury, machine perfusion, metabolomics, lipidomics, extracellular vesicles, cell-free DNA, single-cell sequencing, biomarkers, organ preservation, primary graft dysfunction, and cardiac allograft vasculopathy. Reference lists of relevant articles and recent reviews were manually screened to identify additional publications of interest.

Priority was given to original experimental studies, prospective clinical investigations, randomized clinical trials when available, international consensus statements, and contemporary guidelines from major scientific societies, including the International Society for Heart and Lung Transplantation (ISHLT). High-quality review articles were incorporated primarily to provide a mechanistic context or summarize rapidly evolving fields such as redox biology, mitochondrial function, and molecular phenotyping.

The retrieved evidence was critically evaluated and organized according to the major biological processes that determine donor heart resilience, including oxidative stress, mitochondrial dysfunction, endothelial biology, innate immune activation, regulated cell death, and metabolic adaptation. Particular emphasis was placed on studies investigating emerging molecular technologies—including metabolomics, lipidomics, extracellular vesicle profiling, cell-free nucleic acids, and single-cell approaches—that may complement conventional donor evaluation and enable biological characterization beyond standard clinical assessment.

This review reflects the authors’ critical interpretation of the available evidence and proposes an integrative framework for future research rather than a formal evidence synthesis or quantitative meta-analysis.

### 1.2. Origins of the Donor Heart Redox Phenotype

#### 1.2.1. The Oxidative Biology of the Donor Heart

Donor hearts are traditionally evaluated as isolated clinical entities characterized by chronological age, ventricular function, hemodynamic stability, and laboratory findings obtained immediately before procurement [20,21]. This approach implicitly assumes that the biological condition of the graft is adequately represented by its current physiological state [4]. At the time of transplantation, every donor heart embodies the cumulative consequences of decades of molecular adaptation to intrinsic and extrinsic stressors, many of which leave persistent oxidative, metabolic, and epigenetic imprints that remain clinically invisible [22,23].

We propose the concept of the oxidative biology of the donor heart, referring to the lifelong accumulation of redox-dependent molecular modifications that shape myocardial resilience before transplantation. Oxidative biology develops through cumulative exposure to aging, cardiovascular risk factors, inflammation, environmental influences, and lifestyle, progressively modifying mitochondrial function, endothelial biology, metabolism, and antioxidant capacity [24]. These processes collectively remodel mitochondrial function, endothelial biology, antioxidant defenses, immune responsiveness, and cellular metabolism, ultimately determining the biological reserve available when the heart encounters the profound oxidative challenge of transplantation.

Importantly, oxidative remodeling should not be viewed solely as progressive molecular damage. The final biological phenotype, therefore, reflects the balance between cumulative injury and adaptive resilience rather than the magnitude of oxidative stress alone [25]. Two donor hearts with similar age, ventricular function, and ischemic time may consequently exhibit markedly different capacities to withstand procurement, preservation, and reperfusion because their oxidative biographies differ substantially.

This perspective fundamentally shifts donor assessment from a static evaluation of organ quality toward an appreciation of biological history. In this framework, oxidative biology becomes not merely one component of preservation injury but a continuous determinant of graft vulnerability throughout the lifespan.

#### 1.2.2. Aging and Redox Remodeling

Chronological aging represents the longest and perhaps the most pervasive determinant of the donor heart’s oxidative biography [26]. Even in the absence of overt cardiovascular disease, advancing age is accompanied by gradual deterioration of redox homeostasis characterized by increased mitochondrial reactive oxygen species generation, reduced antioxidant capacity, impaired mitochondrial turnover, accumulation of oxidatively modified proteins, lipid peroxidation, and persistent DNA damage [27]. These alterations evolve over decades and progressively reshape myocardial physiology long before structural abnormalities become clinically apparent.

Mitochondria occupy a central position in this process. Age-dependent impairment of oxidative phosphorylation decreases energetic efficiency while simultaneously increasing electron leakage from the respiratory chain, creating a self-perpetuating cycle of oxidative injury and mitochondrial dysfunction [22]. Reduced mitophagy further promotes the accumulation of dysfunctional mitochondria, amplifying reactive oxygen species production and lowering the threshold for ischemia–reperfusion injury [28]. Consequently, the aged myocardium enters transplantation with diminished metabolic flexibility and reduced capacity to restore energetic homeostasis following reperfusion.

Redox remodeling also profoundly affects the coronary microvasculature [29,30]. Endothelial nitric oxide bioavailability progressively declines because of increased superoxide production, eNOS uncoupling, and oxidation of tetrahydrobiopterin, leading to impaired vasodilatory reserve, endothelial activation, and microvascular dysfunction [31]. These changes compromise tissue perfusion precisely when adequate microcirculatory function becomes essential during graft reperfusion.

Chronological age represents only an imperfect surrogate for these biological processes. Considerable interindividual variability exists in the rate of oxidative remodeling, suggesting that biological age, rather than chronological age, more accurately reflects myocardial resilience [32]. These observations support the assessment of biological rather than chronological age.

Although these determinants are frequently investigated separately, they continuously interact throughout life to shape the biological resilience of the donor myocardium. Their cumulative effects converge on common redox-dependent mechanisms that influence mitochondrial function, endothelial integrity, inflammatory responsiveness, and adaptive reserve. The principal contributors to the donor heart redox phenotype are summarized in Table 1.

#### 1.2.3. Cardiovascular Risk Factors as Chronic Sources of Oxidative Stress

Traditional cardiovascular risk factors contribute to the oxidative biology long before they produce clinically detectable myocardial dysfunction [45]. Hypertension promotes sustained activation of NADPH oxidases, enhances angiotensin II-mediated oxidative signaling, and accelerates endothelial dysfunction through reduced nitric oxide bioavailability [37]. Diabetes mellitus further amplifies oxidative injury by increasing glucose-dependent mitochondrial superoxide production, formation of advanced glycation end-products, activation of protein kinase C signaling, and chronic inflammatory responses [39]. Obesity contributes through adipose tissue inflammation, altered adipokine secretion, insulin resistance, and lipid-mediated mitochondrial dysfunction, whereas dyslipidemia facilitates oxidative modification of lipoproteins, endothelial activation, and vascular inflammation [46]. Cigarette smoking introduces an additional burden of exogenous free radicals and electrophilic compounds that overwhelm endogenous antioxidant systems while directly damaging mitochondrial DNA and vascular endothelium [47]. Consequently, conventional clinical diagnoses may underestimate the degree of biological remodeling already present within the donor myocardium.

#### 1.2.4. Systemic Inflammation and Immunometabolic Reprogramming

Oxidative biology extends beyond classical cardiovascular disease and encompasses persistent interactions between metabolism and the immune system. Chronic low-grade inflammation has emerged as a defining feature of aging and cardiometabolic disorders, creating a biological environment in which oxidative stress and immune activation continuously reinforce one another [48].

Reactive oxygen species not only induce cellular injury but also function as signaling molecules that regulate inflammasome activation, macrophage polarization, cytokine production, and endothelial inflammatory responses [49]. Conversely, activated immune cells generate additional reactive oxygen species through NADPH oxidase activity and mitochondrial metabolism, establishing self-sustaining feedback loops that gradually remodel myocardial tissue [50]. Simultaneously, profound metabolic reprogramming within immune cells alters substrate utilization, mitochondrial function, and redox signaling, promoting persistent inflammatory activation even in the absence of overt infection.

These immunometabolic adaptations influence multiple cellular compartments of the donor heart, including resident macrophages, endothelial cells, fibroblasts, and cardiomyocytes. Rather than representing transient inflammatory episodes, they become integral components of the donor heart’s biological identity, influencing its responses to ischemia, reperfusion, tissue repair, and long-term remodeling after transplantation [48].

#### 1.2.5. Environmental Exposures and the Cardiac Exposome

The donor heart is also shaped by a lifetime of environmental influences that collectively constitute the cardiac exposome [51,52].

Among these factors, chronic exposure to air pollution has emerged as one of the most significant yet frequently overlooked drivers of cardiovascular oxidative injury [53]. Fine particulate matter, nitrogen oxides, ozone, and other airborne pollutants promote systemic oxidative stress, endothelial dysfunction, mitochondrial injury, autonomic imbalance, and chronic vascular inflammation [54]. Although these effects develop gradually, they contribute to persistent remodeling of both the coronary microcirculation and myocardial metabolism, potentially reducing donor hearts’ capacity to tolerate transplantation-associated stress [55,56].

Additional environmental factors—including tobacco smoke, occupational exposures, dietary habits, physical inactivity, psychosocial stress, and circadian rhythm disruption—further modulate myocardial redox homeostasis through interconnected effects on cellular metabolism, immune regulation, mitochondrial function, and antioxidant defense [43,57].

Recognition of the cardiac exposome expands the concept of donor evaluation beyond conventional medical history. Incorporating environmental exposures into donor assessment may improve estimation of the donor heart’s biological resilience beyond conventional clinical variables [58,59].

**Figure 1:**
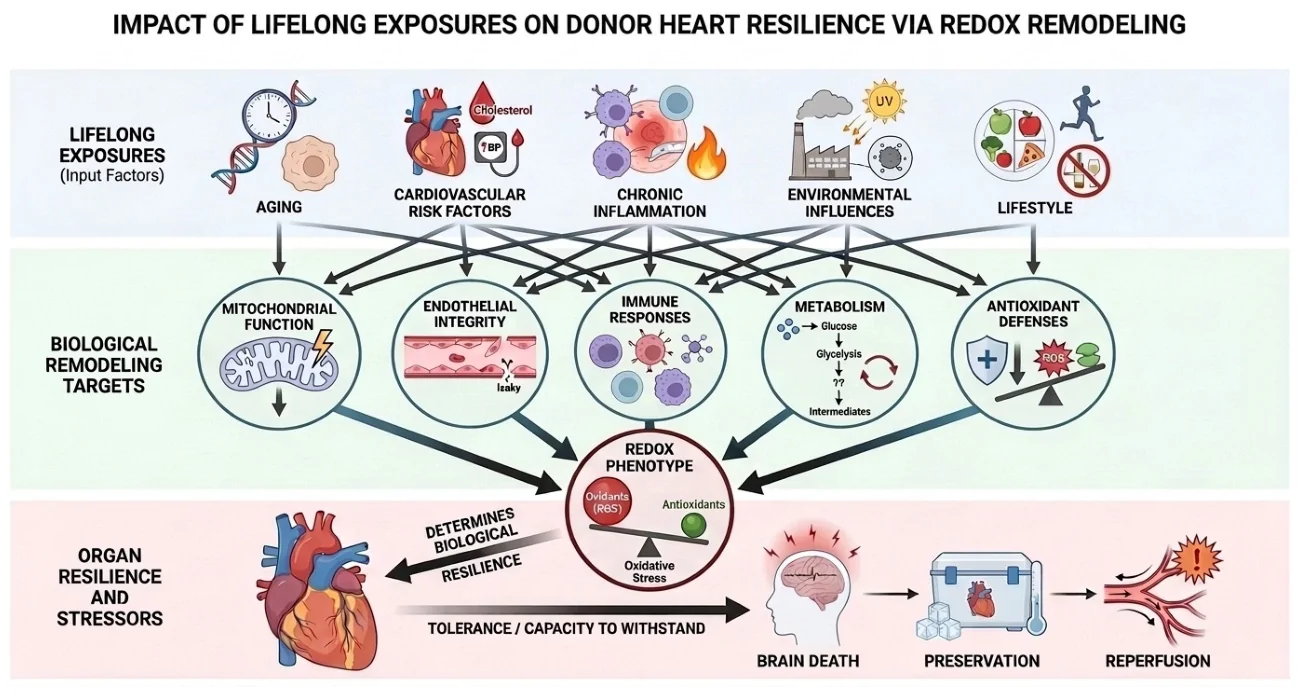
The Oxidative Biography of the Donor Heart. Figure legend: Lifelong exposure to aging, cardiovascular risk factors, chronic inflammation, environmental influences, and lifestyle progressively remodels mitochondrial function, endothelial integrity, immune responses, metabolism, and antioxidant defenses. The resulting redox phenotype determines the biological resilience of the donor heart and its capacity to tolerate brain death, preservation, and reperfusion.

The biological condition of the donor heart is established long before organ procurement through the cumulative effects of aging, cardiometabolic disorders, inflammation, environmental exposures, and lifestyle. Rather than acting independently, these factors progressively remodel multiple redox-sensitive biological systems that ultimately determine myocardial resilience during transplantation. Figure 1 summarizes the lifelong determinants contributing to the development of the donor heart redox phenotype.

### 1.3. Brain Death: The Final Oxidative Hit

The oxidative biography of the donor heart evolves over decades, yet its final chapter is written within hours. Brain death represents a unique biological transition in which chronic oxidative remodeling is abruptly converted into an explosive systemic redox crisis [60,61].

Brain death superimposes acute oxidative, inflammatory, and metabolic stress on the pre-existing donor phenotype [62,63]. Over the course of life, oxidative remodeling develops slowly through cumulative exposure to aging, cardiovascular risk factors, inflammation, and environmental stressors. The donor heart consequently enters transplantation with an entirely different biological phenotype than it possessed only hours earlier.

#### 1.3.1. Catecholamine Storm: Acute Redox Disequilibrium

The sympathetic discharge accompanying brain death constitutes one of the most intense endogenous stress responses encountered in clinical medicine [64,65]. Massive release of catecholamines produces abrupt increases in myocardial oxygen demand, coronary vasoconstriction, intracellular calcium overload, and mechanical stress, thereby rapidly exceeding the myocardium’s metabolic reserve [66,67].

Although transient myocardial dysfunction has long been attributed to catecholamine toxicity, accumulating evidence indicates that oxidative stress is the principal mediator linking sympathetic activation to cellular injury. Excessive β-adrenergic stimulation accelerates mitochondrial electron transport while simultaneously impairing respiratory chain efficiency, increasing electron leakage and reactive oxygen species generation [68]. Activation of NADPH oxidases further amplifies superoxide production, whereas oxidation of tetrahydrobiopterin (BH4) induces endothelial nitric oxide synthase (eNOS) uncoupling, causing the enzyme to generate superoxide rather than nitric oxide and thereby exacerbating oxidative stress [69].

Oxidative modifications of proteins, lipids, and mitochondrial DNA initiate signaling pathways that persist beyond the initial hemodynamic insult, suggesting that the biological consequences of the catecholamine surge extend throughout donor management and may influence subsequent organ preservation and ischemia–reperfusion injury [70,71].

#### 1.3.2. Neurogenic Inflammation: Oxidative Amplification of the Immune Response

Brain death transforms a localized neurological injury into a systemic inflammatory disorder. Disruption of neuroimmune homeostasis initiates widespread cytokine release, complement activation, leukocyte mobilization, and endothelial activation, creating an inflammatory environment that directly interacts with oxidative metabolism [72,73].

Reactive oxygen species generated during this phase are not merely cytotoxic by-products but integral components of inflammatory signaling. Oxidative stress activates redox-sensitive transcription factors, promotes inflammasome assembly, enhances cytokine production, and facilitates leukocyte adhesion to activated vascular endothelium [74,75]. Conversely, infiltrating neutrophils and monocyte-derived macrophages further amplify oxidative stress through NADPH oxidase–dependent respiratory burst activity and myeloperoxidase-mediated generation of reactive oxidants [76]. This reciprocal relationship establishes a self-reinforcing cycle in which inflammation continuously generates oxidative stress while oxidative stress sustains inflammatory activation. By the time donor procurement occurs, the myocardium has already entered a state of coordinated redox-inflammatory activation that may profoundly influence its subsequent response to ischemia and reperfusion.

#### 1.3.3. Mitochondrial Dysfunction: The Biological Point of No Return

Among all intracellular targets affected by brain death, mitochondria occupy a uniquely strategic position because they simultaneously generate reactive oxygen species, regulate cellular energetics, and determine susceptibility to programmed cell death [77,78]. Early mitochondrial dysfunction occurs before prolonged ischemia. Disturbances in calcium homeostasis, excessive adrenergic stimulation, inflammatory mediators, and oxidative stress progressively impair mitochondrial oxidative phosphorylation, reducing ATP production while increasing electron leakage from respiratory chain complexes and mitochondrial reactive oxygen species generation [79]. The loss of mitochondrial quality control further promotes the accumulation of dysfunctional organelles with diminished energetic efficiency and amplified oxidative capacity [80].

These changes fundamentally alter the metabolic behavior of the donor heart. Rather than functioning as an energetically flexible organ capable of adapting to transplantation-associated stress, the myocardium increasingly relies on compromised mitochondrial networks characterized by reduced respiratory reserve and heightened sensitivity to reperfusion-induced injury [81].

#### 1.3.4. Endothelial Injury: Establishing the Microvascular Memory of the Graft

While cardiomyocytes receive much of the attention during donor evaluation, the vascular endothelium may represent one of the earliest and most vulnerable targets of brain death-induced oxidative stress [82]. The vascular endothelium integrates neurohumoral activation, inflammatory signaling, oxidative stress, and coagulation, thereby shaping the microvascular environment of the donor heart before transplantation [83].

Oxidative damage to the endothelial glycocalyx, reduced nitric oxide bioavailability, increased expression of endothelial adhesion molecules, and disruption of intercellular junctions collectively impair microvascular homeostasis before organ retrieval [84]. These alterations promote leukocyte recruitment, platelet activation, increased capillary permeability, and heterogeneous tissue perfusion, creating a persistent microvascular phenotype that may persist through procurement and preservation [85]. Such pre-existing endothelial dysfunction may partly explain why grafts with apparently comparable preservation characteristics often exhibit markedly different reperfusion behavior.

#### 1.3.5. Oxidative Priming Before Procurement

Perhaps the most important consequence of brain death is not the immediate injury it produces but the biological state it creates. We propose the concept of oxidative priming, defined as the establishment of a preconditioned redox environment in which multiple oxidative, metabolic, inflammatory, and endothelial pathways are already activated prior to procurement.

Oxidative priming differs fundamentally from irreversible tissue injury. Rather than representing the extent of accumulated damage, it reflects the readiness of biological systems to respond to subsequent stress. A primed donor heart may therefore exhibit relatively preserved systolic function while simultaneously possessing markedly reduced molecular resilience when exposed to ischemia, preservation, and reperfusion [86].

## 2. Molecular Mechanisms of Preservation Injury

The biological history of the donor heart becomes clinically relevant when the graft is exposed to procurement, preservation, and reperfusion. These insults should not be considered independent of the pre-existing donor phenotype: the same ischemic burden may be well tolerated by one heart and poorly tolerated by another because mitochondrial reserve, antioxidant capacity, endothelial integrity, and metabolic flexibility differ before procurement. Preservation injury therefore provides the functional challenge through which the pre-existing redox phenotype is expressed. Rather than cataloguing all pathways involved in ischemia–reperfusion injury, the following section focuses on the mechanisms most directly relevant to graft resilience and its potential biological assessment.

Ischemia–reperfusion injury represents the principal acute challenge imposed on the pre-existing biological phenotype of the donor heart. During ischemia, interruption of oxidative phosphorylation causes ATP depletion, ionic imbalance, acidosis, and progressive mitochondrial dysfunction. At the same time, succinate accumulates within the ischemic myocardium. Reintroduction of oxygen at reperfusion rapidly restores electron flow, but under these conditions may promote reverse electron transport at mitochondrial complex I and a transient burst of reactive oxygen species. The resulting oxidative stress interacts with calcium overload, mitochondrial permeability transition, lipid peroxidation, and endothelial dysfunction, converting initially reversible metabolic stress into structural cellular injury [87,88].

Their relevance to donor assessment lies in the considerable variability of the myocardial response: comparable ischemic exposure does not necessarily produce comparable graft injury. The extent of injury depends on the metabolic and antioxidant reserve present before procurement and on the capacity of the heart to recover redox homeostasis during reperfusion.

### 2.1. Ischemia–Reperfusion Injury as a Redox Disease

Reperfusion does not merely restore oxygen delivery to an ischemic organ; rather, it exposes the donor heart to an abrupt redox transition, the consequences of which are largely determined by its pre-existing biological state [89,90]. The magnitude of reperfusion injury therefore depends not only on ischemic duration but also on the intrinsic biological resilience of the donor heart and its capacity to restore redox homeostasis [91]. The oxidative mechanisms underlying transplantation are highly dynamic and evolve across successive stages of donor management, organ preservation, and reperfusion. Considering these events as a continuous biological sequence, rather than as isolated processes, facilitates understanding of how cumulative redox disturbances ultimately determine graft viability. The major stages of this progression are summarized in Table 2.

#### 2.1.1. ATP Depletion: Collapse of Energetic Homeostasis

The earliest consequence of ischemia is not oxidative stress itself but interruption of aerobic energy production [74,98]. Oxygen deprivation rapidly suppresses oxidative phosphorylation, forcing cardiomyocytes to rely almost exclusively on anaerobic glycolysis [99].

**Figure 2:**
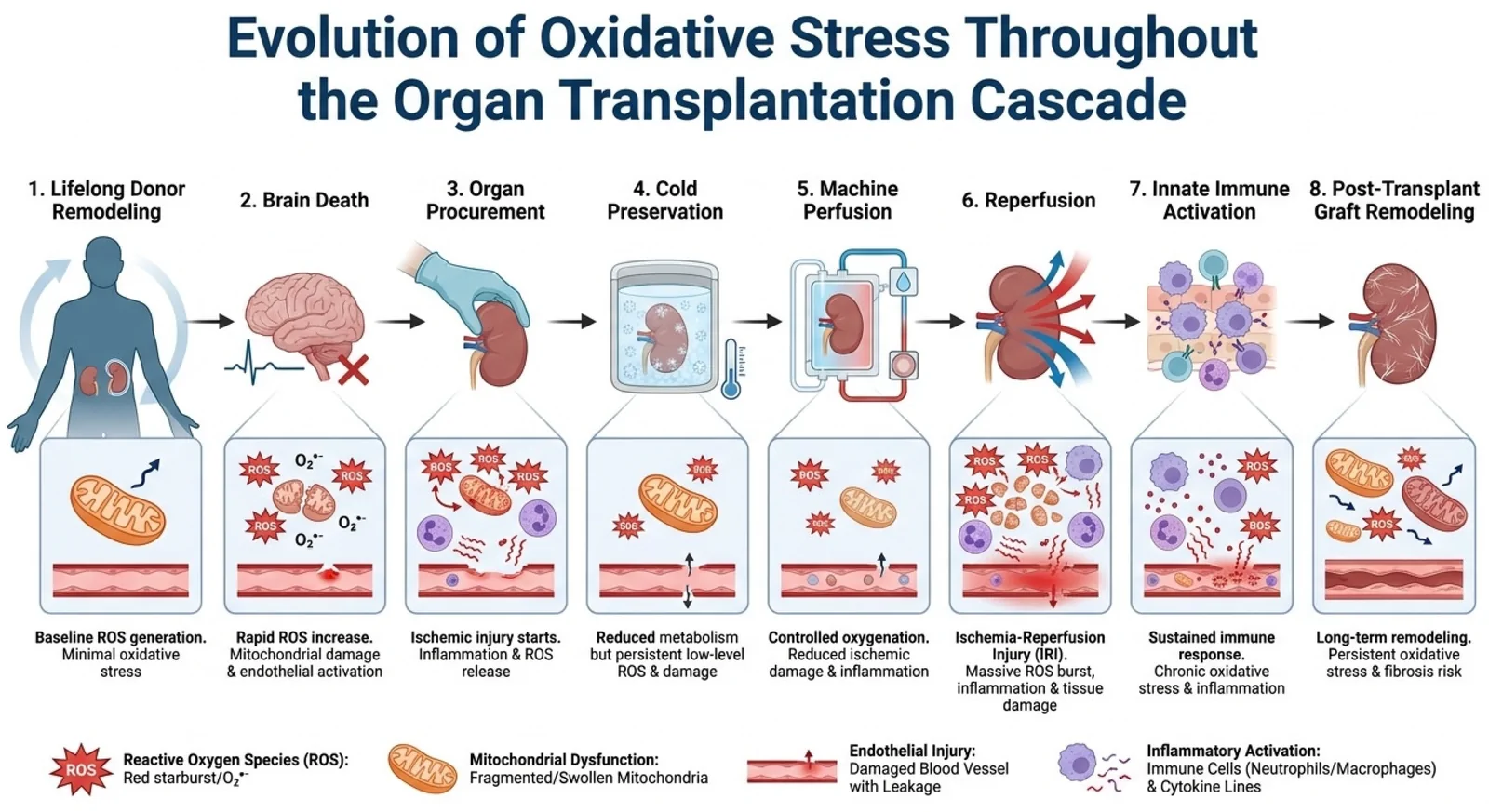
Sequential redox events during heart transplantation. The figure illustrates the evolution of oxidative stress from lifelong donor remodeling through brain death, organ procurement, preservation, machine perfusion, reperfusion, innate immune activation, and post-transplant graft remodeling. Reactive oxygen species, mitochondrial dysfunction, endothelial injury, and inflammatory activation interact continuously throughout this process.

Declining ATP concentrations affect virtually every ATP-dependent cellular process. Failure of ion transporters disrupts sodium and potassium gradients, intracellular pH progressively decreases because of lactate accumulation, and ATP-dependent calcium extrusion becomes increasingly ineffective [100]. Simultaneously, mitochondrial ATP synthase may reverse its direction, hydrolyzing residual ATP to preserve membrane potential and thereby accelerating energetic exhaustion. Although transplantation is often divided into discrete clinical stages, oxidative injury evolves as a continuous biological process extending from donor characteristics to long-term graft remodeling. Figure 2 illustrates the temporal progression of redox disturbances throughout the transplantation pathway.

#### 2.1.2. Succinate Accumulation and Reverse Electron Transport: The Metabolic Switch of Reperfusion

Among the metabolic alterations that develop during ischemia, succinate accumulation has emerged as a central biochemical event linking oxygen deprivation to reperfusion-induced oxidative injury [101,102].

Upon reperfusion, the reintroduction of oxygen enables the rapid oxidation of accumulated succinate by succinate dehydrogenase (complex II), generating a surge of electrons that reduce the ubiquinone pool and drive reverse electron transport through complex I [103]. Under these conditions, electrons are driven backward toward complex I through reverse electron transport, generating an intense burst of mitochondrial superoxide within the first minutes of reperfusion [90].

These findings indicate that reperfusion injury is initiated by metabolic remodeling occurring during ischemia rather than by oxygen restoration alone [104].

#### 2.1.3. Reactive Oxygen Species: From Cellular Signals to Biological Instability

Reactive oxygen species exert a dual role in myocardial biology. Under physiological conditions, they function as essential signaling molecules regulating mitochondrial adaptation, excitation–contraction coupling, autophagy, angiogenesis, and endogenous antioxidant defenses. During reperfusion, however, their magnitude, subcellular localization, and temporal dynamics become profoundly altered, transforming physiological redox signaling into a driver of oxidative injury [105,106]. Loss of spatial and quantitative control transforms physiological redox signaling into oxidative instability [107].

The severity of reperfusion injury depends not only on the magnitude of oxidant generation but also on the capacity of endogenous antioxidant defense systems. Donor hearts with preserved glutathione metabolism, intact thioredoxin-dependent redox regulation, and efficient mitochondrial quality control exhibit greater resilience to reperfusion-induced oxidative stress and are more likely to restore redox homeostasis [108,109].

#### 2.1.4. Calcium Overload: Oxidative Destabilization of Cellular Excitability

Disturbances in calcium homeostasis represent a central mechanism linking ischemia-induced energetic failure to irreversible myocardial injury. During ischemia, progressive ATP depletion impairs ATP-dependent calcium transport, including sarcoplasmic reticulum Ca^2+^ reuptake and sarcolemmal calcium extrusion, while intracellular acidosis transiently attenuates contractile activity by reducing myofilament calcium sensitivity [110]. Reperfusion abruptly disrupts this fragile equilibrium as restoration of physiological pH unmasks calcium-mediated hypercontracture and promotes rapid intracellular and mitochondrial calcium overload [111].

Simultaneously, oxidative modifications of the ryanodine receptor (RyR2), sarcoplasmic reticulum Ca^2+^-ATPase (SERCA2a), sodium–calcium exchanger (NCX), and mitochondrial calcium uniporter further disrupt intracellular calcium homeostasis, promoting cytosolic and mitochondrial calcium overload [112]. Elevated mitochondrial calcium stimulates mitochondrial reactive oxygen species production, whereas oxidative modification of calcium-handling proteins further impairs calcium homeostasis, creating a self-amplifying cycle of calcium overload and oxidative stress [113]. Oxidative stress and calcium overload therefore amplify one another, accelerating irreversible injury.

#### 2.1.5. Mitochondrial Permeability Transition: Commitment to Irreversible Injury

The opening of the mitochondrial permeability transition pore (mPTP) is the critical event at which reversible metabolic dysfunction progresses to irreversible cell injury. Rather than constituting an isolated molecular event, mPTP opening integrates the cumulative consequences of ATP depletion, succinate oxidation, reactive oxygen species generation, calcium overload, and mitochondrial membrane destabilization, ultimately leading to mitochondrial depolarization, bioenergetic collapse, and activation of regulated cell death pathways [114,115]. At this stage, restoration of coronary perfusion is insufficient to reverse mitochondrial dysfunction, as the bioenergetic and redox regulatory systems required to maintain cellular homeostasis have become irreversibly impaired.

### 2.2. Redox Homeostasis as the Master Regulator of Cell Fate

Growing evidence indicates that these pathways are extensively interconnected and frequently converge on shared mechanisms, including mitochondrial dysfunction, oxidative stress, calcium dysregulation, and inflammatory signaling [116]. Within this interconnected network of regulated cell death, oxidative stress serves as a central organizing mechanism rather than a single upstream trigger. Reactive oxygen species coordinate multiple stress-response pathways by modulating mitochondrial metabolism, iron handling, membrane integrity, inflammasome activation, DNA damage responses, endoplasmic reticulum stress, and innate immune signaling [117]. Consequently, cell fate is determined not simply by the amount of reactive oxygen species generated but by their subcellular distribution, temporal pattern of production, and the ability of endogenous antioxidant systems to preserve redox homeostasis.

This concept may also explain one of the most consistent observations in transplantation biology: donor hearts exposed to apparently comparable ischemic conditions frequently exhibit markedly different patterns of tissue injury. The determining factor may not be which death pathway is activated, but rather the myocardium’s intrinsic ability to maintain redox homeostasis amid competing stress-response programs.

#### 2.2.1. Apoptosis: Programmed Adaptation to Oxidative Injury

Among the regulated cell death pathways, apoptosis represents the most highly regulated and energy-dependent response to oxidative stress [118]. Moderate oxidative injury promotes BAX/BAK-mediated mitochondrial outer membrane permeabilization, resulting in cytochrome c release, apoptosome formation, and caspase activation while largely preserving plasma membrane integrity and minimizing inflammatory signaling.

#### 2.2.2. Necroptosis: Oxidative Failure of Cellular Integrity

Necroptosis [119] arises when oxidative injury occurs in a biological environment that can no longer sustain controlled apoptotic signaling. Activation of receptor-interacting protein kinases and MLKL disrupts plasma membrane integrity, leading to the abrupt release of intracellular constituents that amplify sterile inflammation [120].

#### 2.2.3. Ferroptosis: Collapse of the Lipid Redox Barrier

Among all forms of regulated cell death, ferroptosis most directly reflects failure of redox homeostasis [121]. Unlike apoptosis or necroptosis, ferroptosis is fundamentally driven by uncontrolled lipid peroxidation resulting from disruption of glutathione metabolism, glutathione peroxidase 4 activity, iron handling, and phospholipid antioxidant defenses [122].

The donor heart contains exceptionally abundant mitochondria and highly oxidative membrane phospholipids, creating conditions in which even modest disturbances of antioxidant capacity may trigger extensive lipid oxidation [123]. Consequently, ferroptosis may function less as an isolated death program than as an indicator that antioxidant buffering has become insufficient to preserve membrane integrity. Rather than representing the final stage of oxidative injury, ferroptosis may therefore serve as one of the earliest manifestations of irreversible redox disequilibrium within the transplanted myocardium.

#### 2.2.4. Pyroptosis: When Oxidative Stress Becomes Inflammatory

Persistent mitochondrial dysfunction promotes the release of mitochondrial DNA, oxidized lipids, ATP, and other danger-associated molecular patterns that activate inflammasomes and initiate pyroptotic signaling [124].

Unlike apoptosis, pyroptosis transforms individual cellular injury into coordinated inflammatory activation. Formation of membrane pores by gasdermin proteins enables the rapid release of IL-1β, IL-18, and other inflammatory mediators that recruit innate immune cells and amplify oxidative stress throughout the graft.

#### 2.2.5. Immunogenic Cell Death: Translating Oxidative Injury into Alloimmunity

Perhaps the most clinically relevant consequence of oxidative stress during transplantation is its ability to modify the immunological meaning of cell death [125].

**Figure 3:**
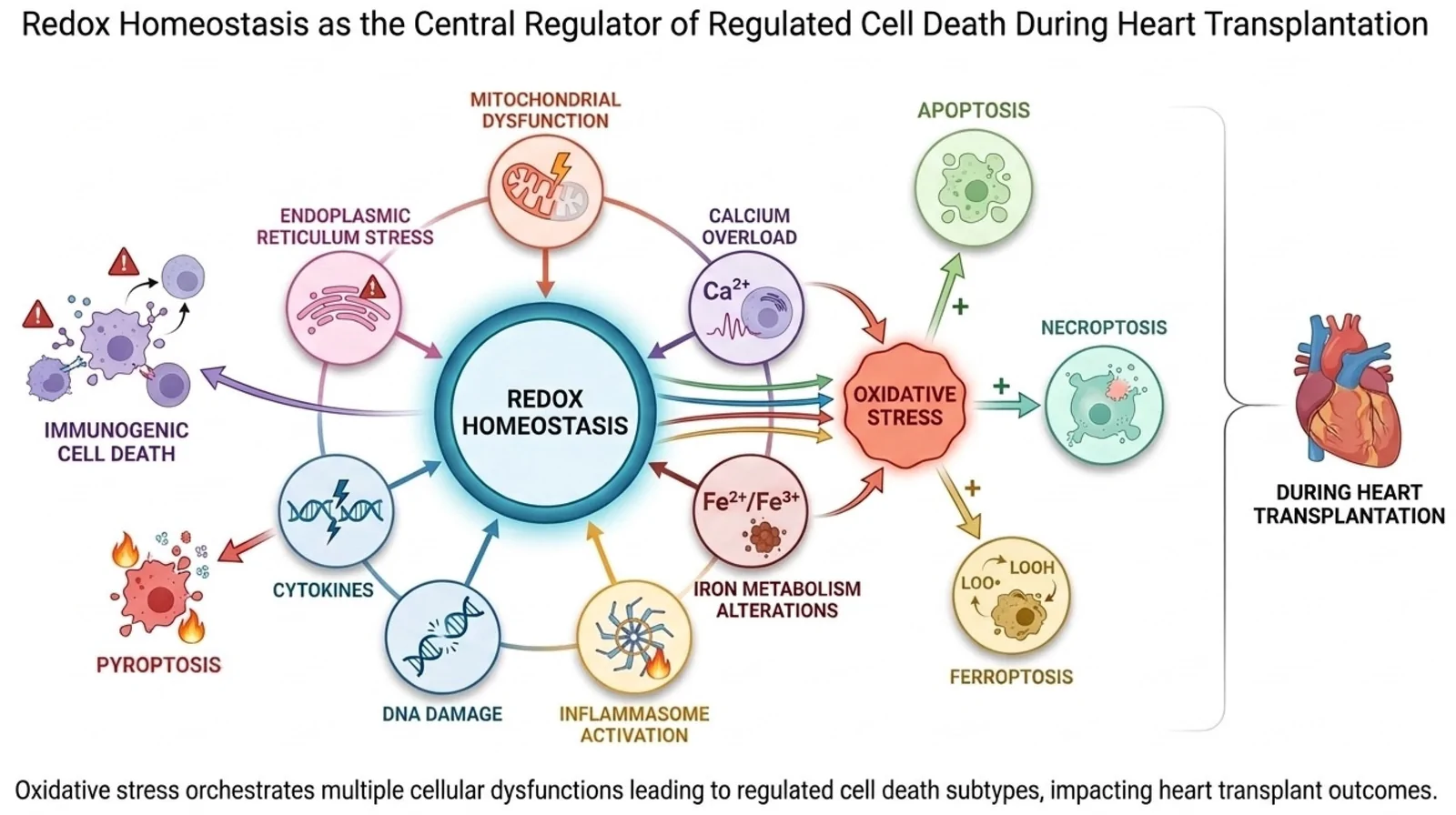
Redox homeostasis as the central regulator of regulated cell death. Oxidative stress integrates mitochondrial dysfunction, calcium overload, iron metabolism, inflammasome activation, DNA damage, and endoplasmic reticulum stress into a coordinated network that regulates apoptosis, necroptosis, ferroptosis, pyroptosis, and immunogenic cell death during heart transplantation.

Oxidative modifications of proteins, lipids, nucleic acids, and extracellular matrix components profoundly influence the repertoire of danger signals released from injured cells. Exposure of calreticulin, extracellular ATP release, HMGB1 secretion, oxidized mitochondrial DNA, and lipid peroxidation products act as danger-associated molecular patterns that link oxidative injury to innate immune activation, thereby promoting antigen presentation and subsequent adaptive alloimmune responses [126,127]. These interconnected redox-dependent pathways are summarized in Figure 3.

### 2.3. Endothelial Redox Biology: The Endothelium as the Biological Interpreter of Oxidative Stress

The vascular endothelium integrates oxidative, inflammatory, thrombotic, and metabolic signals generated during ischemia–reperfusion [128,129].

The biological consequences of oxidative stress depend less on the absolute amount of reactive oxygen species than on endothelial cells’ ability to maintain redox homeostasis and adaptive signaling despite profound metabolic disturbance [130]. Endothelial dysfunction, therefore, contributes directly to primary graft dysfunction and chronic graft remodeling by coordinating vascular, immune, and metabolic responses [131,132].

#### 2.3.1. Glycocalyx Injury: Loss of the Endothelium’s Redox Sensor

The endothelial glycocalyx is the first endothelial structure exposed to blood-borne reactive species and inflammatory mediators entering the coronary circulation after reperfusion, serving as an essential interface between the circulating blood and the vascular wall [133,134].

Oxidative stress rapidly fragments glycocalyx components by activating matrix-degrading enzymes and directly modifying proteoglycans and glycosaminoglycans [135]. Loss of glycocalyx integrity compromises endothelial mechanotransduction and impairs restoration of vascular homeostasis after reperfusion.

#### 2.3.2. eNOS Uncoupling: From Nitric Oxide Signaling to Oxidative Amplification

Under physiological conditions, nitric oxide integrates vasodilation, mitochondrial respiration, platelet inhibition, leukocyte quiescence, and endothelial survival into a coordinated homeostatic program [136]. During ischemia–reperfusion, oxidation of tetrahydrobiopterin and disruption of intracellular redox balance uncouple endothelial nitric oxide synthase, converting an enzyme designed to suppress oxidative stress into an additional source of superoxide production [137].

#### 2.3.3. Microvascular Dysfunction: When Reperfusion Fails at the Tissue Level

Successful transplantation restores epicardial coronary flow within minutes, yet restoration of macroscopic perfusion does not necessarily translate into effective oxygen delivery at the level of individual cardiomyocytes. The discrepancy between coronary patency and tissue oxygenation reflects the biological state of the coronary microcirculation [138]. Oxidative stress profoundly alters microvascular behavior through endothelial swelling, glycocalyx degradation, impaired nitric oxide signaling, platelet activation, leukocyte adhesion, capillary obstruction, and heterogeneous vasomotor regulation [139]. These abnormalities produce a highly fragmented perfusion pattern in which adjacent myocardial regions may simultaneously experience hyperemia, adequate perfusion, or persistent ischemia.

This spatial heterogeneity may represent one of the principal determinants of regional myocardial injury, mitochondrial recovery, and subsequent ventricular remodeling [140].

#### 2.3.4. Leukocyte Recruitment: Endothelial Control of Sterile Inflammation

Recruitment of circulating leukocytes during reperfusion has classically been interpreted as a consequence of inflammatory cytokine release. Contemporary evidence, however, indicates that endothelial redox signaling, glycocalyx disruption, complement activation, and damage-associated molecular patterns (DAMPs) are equally important determinants of leukocyte recruitment and activation [141]. Growing evidence suggests a more nuanced mechanism whereby endothelial cells actively regulate both the magnitude and cellular composition of the inflammatory infiltrate through redox-dependent expression of adhesion molecules, chemokines, and immunomodulatory mediators [142].

#### 2.3.5. Endothelial–Immune Crosstalk: The Gateway to Chronic Graft Remodeling

The biological significance of endothelial dysfunction extends far beyond the immediate reperfusion period. Persistent disturbances in endothelial redox homeostasis establish long-lasting communication networks linking vascular cells, resident immune populations, circulating leukocytes, platelets, fibroblasts, and cardiomyocytes [138].

Within this multicellular environment, oxidative stress is propagated through reciprocal signaling rather than direct diffusion of reactive oxygen species alone [143,144]. Cardiac allograft vasculopathy, diffuse interstitial fibrosis, and progressive endothelial dysfunction may therefore be viewed not as isolated pathological entities but as interconnected manifestations of persistent endothelial injury in which failure to restore redox homeostasis represents a central mechanistic contributor after transplantation [145,146].

Preservation of endothelial redox competence may therefore represent one of the most promising therapeutic targets for improving donor heart resilience and long-term graft function. It is not merely the first tissue exposed to oxidative stress after reperfusion; it is the biological interface that determines whether oxidative signals are resolved through adaptive vascular homeostasis or amplified into sustained inflammation, microvascular failure, and chronic graft remodeling [147].

**Figure 4:**
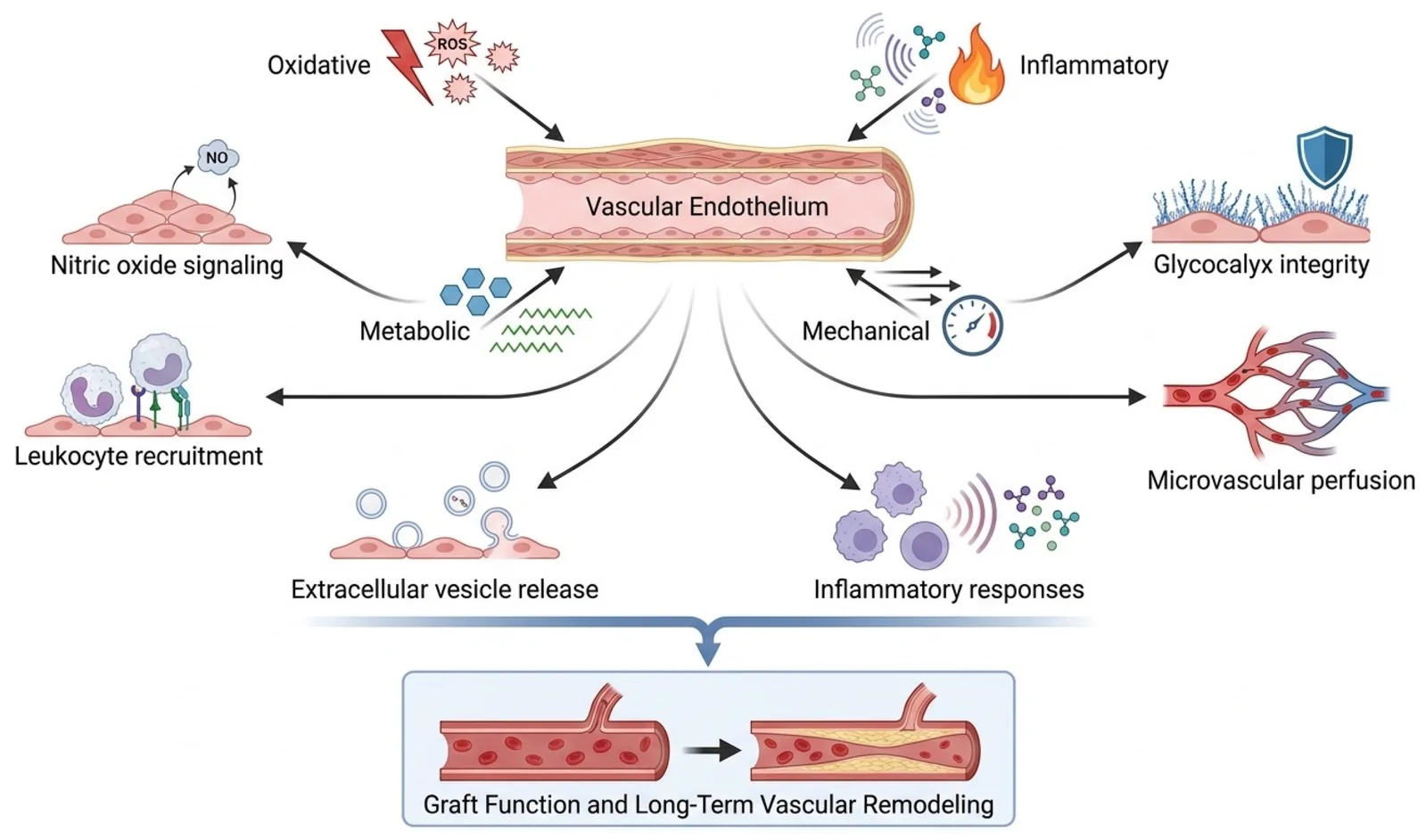
Endothelial integration of oxidative stress during heart transplantation. Oxidative, inflammatory, metabolic, and mechanical stimuli converge on the vascular endothelium, regulating nitric oxide signaling, glycocalyx integrity, leukocyte recruitment, microvascular perfusion, extracellular vesicle release, and inflammatory responses, collectively determining graft function and long-term vascular remodeling.

The vascular endothelium functions as the principal biological interface translating oxidative stress into vascular, inflammatory, and metabolic responses [139]. Rather than representing a passive target of injury, endothelial cells coordinate the tissue-wide consequences of reperfusion. Figure 4 summarizes this central regulatory role.

## 3. Redox Phenotyping

### 3.1. Mitochondria as the Biological Memory and Decision Center of the Donor Heart

Contemporary mitochondrial biology suggests that they function simultaneously as metabolic sensors, redox regulators, mechanochemical transducers, innate immune platforms, and determinants of cellular fate [148,149]. Their role extends well beyond energy production; they continuously integrate information originating from aging, metabolism, inflammation, environmental exposures, ischemia, and mechanical stress into coordinated adaptive responses.

From this perspective, mitochondria constitute the biological archive of the donor heart. Every episode of oxidative stress, metabolic adaptation, inflammatory activation, or environmental exposure leaves persistent molecular signatures within the mitochondrial network [111,150]. In many respects, mitochondrial function represents the biological denominator through which all determinants of donor quality ultimately converge.

#### 3.1.1. Energetic Failure: Loss of Adaptive Metabolism Rather than ATP Alone

Healthy myocardium possesses remarkable metabolic plasticity, continuously adjusting substrate utilization according to oxygen availability, hormonal signaling, workload, and nutrient supply [151,152]. During donor management and transplantation, however, this flexibility progressively deteriorates. Aging, cardiovascular disease, systemic inflammation, brain death, and ischemia collectively narrow the metabolic repertoire available to the myocardium, rendering oxidative metabolism increasingly rigid and energetically inefficient.

Consequently, reperfusion challenges not only ATP production but also the mitochondrial network’s ability to rapidly reorganize energy metabolism under conditions of profound biological stress [153,154]. Hearts with preserved metabolic adaptability may restore energetic homeostasis despite substantial ischemic injury, whereas those with limited metabolic reserve frequently progress toward irreversible dysfunction despite apparently acceptable preservation conditions.

#### 3.1.2. Oxidative Phosphorylation: The Central Regulator of Redox Homeostasis

Beyond ATP generation, oxidative phosphorylation maintains intracellular redox homeostasis by coordinating oxygen utilization, electron transport, NADH oxidation, and antioxidant regeneration [155,156]. The respiratory chain continuously regulates the relationship between oxygen utilization, electron transport, nicotinamide adenine dinucleotide oxidation, reactive oxygen species generation, and antioxidant regeneration [157,158].

This integrated function explains why disturbances in oxidative phosphorylation have consequences that extend far beyond energetic insufficiency. Even subtle alterations in respiratory efficiency modify mitochondrial redox signaling, calcium handling, metabolite exchange, and activation of stress-response pathways throughout the cell [159,160]. The biological significance of oxidative phosphorylation, therefore, lies not only in its ability to generate ATP but also in its capacity to preserve coordinated communication between metabolism and redox regulation.

#### 3.1.3. Mitophagy: Continuous Quality Control of the Mitochondrial Population

Unlike most cellular organelles, mitochondria exist as dynamic populations characterized by continuous turnover [161]. By continuously renewing the mitochondrial network, mitophagy preserves respiratory efficiency, limits excessive reactive oxygen species generation, prevents the release of pro-inflammatory mitochondrial components, and maintains metabolic adaptability under changing physiological conditions [162].

During transplantation, this quality-control system becomes progressively compromised. Oxidative injury, ATP depletion, and inflammatory signaling impair mitophagic flux precisely when mitochondrial renewal becomes most critical. Dysfunctional organelles therefore accumulate, generating disproportionate amounts of reactive oxygen species while simultaneously losing energetic competence [163].

#### 3.1.4. Mitochondrial Dynamics: Architectural Determinants of Biological Resilience

Under physiological conditions, coordinated mitochondrial fusion and fission maintain mitochondrial quality control. Fusion enables functional complementation through the exchange of matrix contents and mitochondrial DNA, whereas fission isolates dysfunctional mitochondrial regions for selective elimination by mitophagy, thereby preserving respiratory competence and metabolic flexibility [164]. Transplantation profoundly perturbs this equilibrium. Excessive oxidative stress favors pathological mitochondrial fragmentation, disrupting communication within the network and limiting metabolic cooperation between adjacent organelles. Fragmented mitochondria generate more reactive oxygen species, exhibit diminished respiratory efficiency, and become increasingly susceptible to permeability transition [165,166]. Conversely, impaired fusion restricts redistribution of functional mitochondrial components required for recovery following reperfusion.

#### 3.1.5. Mitochondrial DNA Signaling: From Metabolic Disturbance to Innate Immunity

The biological influence of mitochondria extends beyond intracellular metabolism into the regulation of innate immunity. Mitochondrial DNA retains structural characteristics inherited from its bacterial evolutionary origin and therefore functions as a potent danger-associated molecular pattern when released into inappropriate cellular compartments [167,168].

Oxidative injury destabilizes mitochondrial membranes, facilitating the escape of mitochondrial DNA into the cytosol and extracellular environment [169,170]. Recognition by pattern-recognition receptors initiates interferon signaling, inflammasome activation, cytokine production, and amplification of sterile inflammation within the transplanted heart. Damaged mitochondria are not merely metabolically inefficient; they become active immunological organelles capable of converting disturbances in energy metabolism into persistent inflammatory signaling.

### 3.2. From Biomarkers to Biological Fingerprints: Defining the Redox Phenotype of the Donor Heart

For decades, transplantation research has pursued an ideal biomarker to predict donor heart viability before implantation. Numerous circulating molecules have been proposed, including markers of myocardial injury, oxidative stress, endothelial dysfunction, inflammation, and mitochondrial damage [171]. Although many demonstrate statistical associations with graft function, very few have achieved meaningful clinical utility. This persistent translational gap may reflect a more fundamental problem. The biological condition of the donor heart is unlikely to be captured by any individual molecule because viability is not a single molecular property but an emergent characteristic arising from interactions among multiple adaptive systems.

No single biomarker adequately reflects the complexity of donor heart biology. Instead, complementary molecular signatures derived from multiple biological domains provide a more comprehensive representation of oxidative adaptation and biological resilience. Representative biomarkers that may contribute to future clinical redox phenotyping are summarized in Table 3.

#### 3.2.1. Oxidative Stress Biomarkers: Measuring the Consequences Rather than the Process

Conventional oxidative stress biomarkers—including lipid peroxidation products, oxidized proteins, nucleic acid oxidation products, and reactive oxygen species-derived metabolites—have substantially advanced understanding of transplantation biology [190]. They provide important evidence that oxidative injury accompanies donor management, preservation, and reperfusion.

Nevertheless, these molecules primarily represent biochemical footprints of oxidative reactions that have already occurred. They quantify accumulated molecular damage but offer limited insight into the dynamic mechanisms that regulate oxidative homeostasis. Oxidative injury is therefore more accurately regarded as the historical record of preceding biological events than as a direct measure of current mitochondrial competence or future adaptive potential.

#### 3.2.2. Antioxidant Capacity: The Forgotten Dimension of Donor Assessment

The antioxidant network extends far beyond individual enzymes. It encompasses glutathione metabolism, thioredoxin systems, peroxiredoxins, superoxide dismutases, catalase, mitochondrial NADPH regeneration, Nrf2-dependent transcriptional programs, and numerous metabolic pathways that maintain intracellular reducing potential [191]. Together, these mechanisms determine whether oxidative disturbances remain transient physiological signals or evolve into irreversible molecular injury. Assessment of antioxidant competence represents measurement of biological reserve rather than biochemical damage.

#### 3.2.3. Metabolomics: Reading the Functional State of the Mitochondrial Network

Among emerging technologies, metabolomics provides perhaps the closest approximation of real-time biological function. Unlike genomic and proteomic analyses, which primarily characterize biological composition and potential, metabolomic profiling captures the tissue’s functional metabolic phenotype by reflecting the integrated effects of mitochondrial activity, substrate utilization, enzymatic flux, and environmental influences at the time of sampling [192].

Within donor hearts, alterations involving tricarboxylic acid cycle intermediates, amino acids, acylcarnitines, purine metabolism, lactate, and redox cofactors collectively describe the energetic behavior of the myocardium during preservation and reperfusion [193]. Metabolomics, therefore, represents considerably more than biomarker discovery. It provides a functional portrait of the donor heart at the precise moment transplantation decisions are made.

#### 3.2.4. Lipidomics: Decoding Membrane Vulnerability

Lipids are frequently considered passive structural components of cellular membranes. Oxidative modification of phospholipid species alters membrane fluidity, mitochondrial architecture, receptor signaling, and production of numerous bioactive lipid mediators [194]. Simultaneously, remodeling of cardiolipins and other mitochondrial phospholipids directly influences the organization of the respiratory chain and its susceptibility to permeability transition.

#### 3.2.5. Extracellular Vesicles: Circulating Carriers of the Redox Phenotype

Extracellular vesicles are active biological messengers transporting proteins, lipids, metabolites, mitochondrial components, microRNAs, and other regulatory molecules between cells [195]. The molecular cargo of extracellular vesicles reflects the physiological state of their cell of origin while simultaneously influencing the biology of recipient cells [196,197]. During donor management and reperfusion, endothelial cells, cardiomyocytes, immune cells, and platelets release distinct vesicle populations carrying redox-sensitive molecular signatures capable of propagating inflammatory activation, endothelial dysfunction, or adaptive repair.

#### 3.2.6. Cell-Free DNA: Molecular Evidence of Biological Instability

The release of cell-free nuclear and mitochondrial DNA into circulation has emerged as one of the most promising indicators of tissue injury [198]. Particularly relevant is mitochondrial DNA, whose bacterial evolutionary origin renders it a potent activator of innate immune pathways [199]. Elevated circulating mitochondrial DNA, therefore, reflects more than mitochondrial disruption alone. It signifies conversion of metabolic injury into inflammatory signaling, establishing a direct mechanistic connection between oxidative stress and sterile immune activation. Measurement of cell-free DNA may thus identify donor hearts approaching a critical threshold at which metabolic dysfunction begins to generate sustained immunological consequences.

#### 3.2.7. Multi-Omic Redox Signatures: Toward Systems Biology of Donor Heart Viability

The future of precision transplantation will likely depend on integrating multiple layers of biological information into comprehensive molecular fingerprints. Oxidative biomarkers define cumulative molecular injury; antioxidant profiling quantifies adaptive reserve; metabolomics captures ongoing energetic activity; lipidomics characterizes membrane remodeling; extracellular vesicles reveal intercellular communication; and cell-free nucleic acids reflect structural and immunological instability [200]. Considered independently, each platform provides only partial information. Integrated computationally, however, they begin to reconstruct the multidimensional biological state of the donor heart.

Such multidimensional biological fingerprints provide the logical foundation for the next generation of donor heart assessment, in which transplantation decisions are informed not by isolated variables but by quantitative characterization of the organ’s adaptive capacity.

### 3.3. Dynamic Redox Phenotyping During Machine Perfusion

Conventional donor assessment is performed before the biological consequences of transplantation become fully manifest. Echocardiography, hemodynamic measurements, laboratory investigations, and static imaging estimate organ quality under donor conditions but provide limited information regarding how the heart will respond to ischemia, reperfusion, and implantation. In contrast, machine perfusion allows direct observation of graft biology while the organ remains outside the body, creating an entirely new opportunity to evaluate functional resilience rather than infer it from surrogate clinical variables [201,202].

Static preservation interrupts biological activity and, therefore, limits assessment to measurements obtained before procurement. Machine perfusion maintains cellular metabolism, mitochondrial respiration, vascular function, and tissue viability, enabling continuous interrogation of biological processes that were previously inaccessible [203,204]. The donor heart becomes an active biological system in which metabolic adaptation, oxidative stress, endothelial function, and inflammatory activation can be monitored in real time as they evolve.

This temporal dimension distinguishes dynamic phenotyping from conventional donor evaluation and provides an opportunity to identify biological trajectories associated with either successful recovery or progressive deterioration.

#### 3.3.1. Normothermic Machine Perfusion: Preserving Biological Activity

Normothermic machine perfusion maintains the donor organ in a metabolically active state by restoring physiological temperature and continuous coronary perfusion [205,206]. Unlike static cold storage, which suppresses cellular metabolism, normothermic perfusion preserves mitochondrial respiration, substrate utilization, endothelial signaling, and myocardial contractility, thereby more closely approximating the physiological environment [207].

The principal advantage of this approach lies not simply in extending preservation time but in maintaining active biological processes that can be interrogated during preservation [208]. Mitochondrial function, myocardial oxygen consumption, coronary vascular resistance, lactate kinetics, and contractile performance become measurable variables rather than inferred physiological properties.

#### 3.3.2. Hypothermic Oxygenated Perfusion: Supporting Mitochondrial Recovery

Hypothermic oxygenated perfusion adopts a fundamentally different biological strategy. Rather than sustaining metabolic homeostasis, it minimizes metabolic demand while preserving mitochondrial oxygen availability [209,210]. This approach limits ATP depletion, attenuates succinate accumulation, and supports restoration of oxidative phosphorylation before normothermic reperfusion.

One experimental study [211] indicated that oxygen delivery during hypothermia facilitates partial recovery of mitochondrial respiratory function without exposing the myocardium to the abrupt metabolic transition associated with immediate warm reperfusion. Consequently, hypothermic oxygenated perfusion may reduce the intensity of the initial oxidative burst and preserve mitochondrial integrity before implantation. From a redox perspective, hypothermic perfusion should therefore be viewed as a strategy to stabilize mitochondrial metabolism rather than simply to prolong preservation.

#### 3.3.3. Real-Time Metabolic Monitoring

One of the most important advances introduced by machine perfusion is the ability to monitor metabolism continuously rather than retrospectively. Sequential assessment of oxygen consumption, lactate production and clearance, glucose utilization, acid-base balance, high-energy phosphate metabolism, and selected metabolites provides insight into the evolving metabolic behavior of the donor heart throughout preservation [212].

Metabolic trajectories frequently provide more informative signals than isolated measurements. Progressive normalization of lactate metabolism, stabilization of oxygen extraction, or recovery of substrate utilization may indicate restoration of mitochondrial function, whereas persistent metabolic instability suggests failure of adaptive recovery despite apparently preserved contractile performance [213].

#### 3.3.4. Functional Redox Assessment and Continuous Biomarker Monitoring

**Figure 5:**
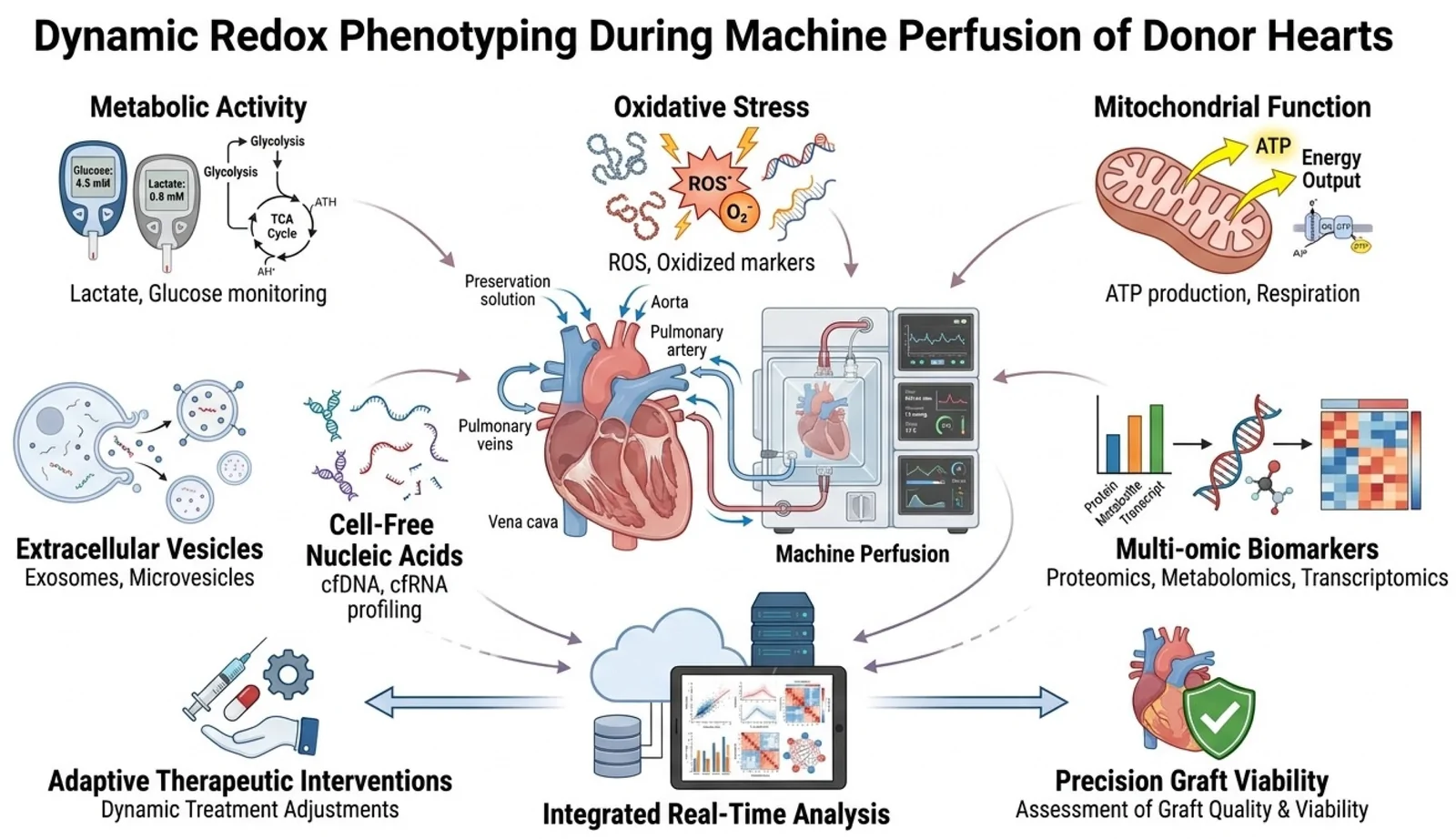
Dynamic Redox Phenotyping During Machine Perfusion. Continuous assessment of metabolic activity, oxidative stress, mitochondrial function, extracellular vesicles, cell-free nucleic acids, and multi-omic biomarkers enables real-time characterization of donor heart biology. Integration of these data supports adaptive therapeutic interventions and precision assessment of graft viability before transplantation.

Biological resilience depends not only on the extent of oxidative injury but also on the graft’s capacity to restore redox homeostasis during preservation. Machine perfusion enables this capacity to be assessed dynamically through serial measurements of mitochondrial respiration, oxygen utilization, lactate kinetics, glutathione turnover, redox-sensitive metabolites, endothelial responses, and circulating molecular markers [214,215,216]. Repeated analysis of oxidative stress markers, extracellular vesicles, cell-free nucleic acids, inflammatory mediators, and metabolic intermediates may therefore define biological trajectories rather than isolated biochemical states [217]. Integration of these complementary signals could identify recovery or progressive loss of graft resilience before deterioration becomes apparent from conventional hemodynamic or contractile parameters [60,218]. Thus, machine perfusion provides a platform for functional redox phenotyping, in which graft viability is evaluated by its capacity to maintain or restore biological homeostasis during preservation. Figure 5 illustrates how real-time biological monitoring may enable adaptive redox phenotyping throughout preservation.

## 4. Therapeutic Opportunities

### 4.1. Redox Engineering of the Donor Heart: From Cytoprotection to Biological Reprogramming

Experimental antioxidant strategies have shown substantial efficacy, but clinical translation remains limited [219,220]. Because reactive oxygen species also serve essential physiological signaling functions, therapeutic strategies should aim to restore redox homeostasis rather than indiscriminately suppress oxidant generation [221,222]. Ex vivo preservation provides a particularly attractive therapeutic window in which mitochondrial, metabolic, endothelial, and cellular stress-response pathways can be selectively modulated before implantation. Representative approaches and their translational status are summarized in Table 4.

#### 4.1.1. Reinforcing Endogenous Antioxidant Networks

The limited clinical success of conventional antioxidant therapy has fundamentally reshaped our understanding of redox biology by demonstrating that endogenous antioxidant defenses function as integrated adaptive signaling networks rather than as collections of independent radical-scavenging molecules [111,238]. Consequently, the preservation of redox homeostasis depends on the coordinated regulation of multiple interconnected pathways rather than on the augmentation of a single antioxidant species. Enzymatic defenses, glutathione metabolism, thioredoxin systems, peroxiredoxins, and redox-sensitive transcriptional pathways cooperate to preserve intracellular homeostasis across multiple cellular compartments.

Accordingly, contemporary therapeutic strategies increasingly aim to reinforce intrinsic antioxidant capacity rather than directly scavenging reactive oxygen species [239]. Supporting endogenous defense mechanisms allows physiological redox signaling to remain intact while preventing the transition toward uncontrolled oxidative injury.

#### 4.1.2. Mitochondrial Redox Modulation

Because mitochondria are the principal sites of both energy production and the regulated generation of reactive oxygen species, selective modulation of mitochondrial redox homeostasis has emerged as one of the most promising approaches for donor heart preservation [240]. Mitochondria-targeted antioxidants, stabilization of respiratory chain function, preservation of cardiolipin integrity, and enhancement of mitochondrial quality control collectively seek to restore physiological electron flux through the respiratory chain, preserve mitochondrial bioenergetic efficiency, and prevent pathological electron leakage that drives oxidative injury, rather than merely scavenging reactive oxygen species after they have been generated [241].

Restoration of mitochondrial function may simultaneously improve ATP synthesis, calcium homeostasis, metabolic flexibility, innate immune regulation, and cell survival, thereby targeting several determinants of graft viability through a common upstream mechanism [242,243].

#### 4.1.3. Metabolic Reprogramming of Reperfusion

Recognition of succinate-driven reverse electron transport has shifted attention toward the metabolic events preceding reperfusion [244]. Rather than treating oxidative stress after it develops, interventions targeting succinate metabolism aim to modify the biochemical conditions that underlie the initial burst of mitochondrial reactive oxygen species.

Targeting succinate metabolism represents a shift from downstream antioxidant therapy toward upstream metabolic intervention. By modulating mitochondrial substrate utilization, preserving respiratory efficiency, and limiting pathological metabolite accumulation during ischemia, these approaches aim to prevent reverse electron transport and the ensuing burst of mitochondrial reactive oxygen species at the onset of reperfusion, rather than scavenging reactive oxygen species after oxidative injury has already begun [245,246].

#### 4.1.4. Preserving Membrane Redox Integrity

The growing recognition of ferroptosis has reinforced the concept that oxidative injury is fundamentally linked to membrane biology [247]. Consequently, the preservation of phospholipid integrity, the maintenance of glutathione-dependent phospholipid repair systems, the regulation of intracellular iron homeostasis, and the stabilization of mitochondrial membrane composition have emerged as complementary strategies for preserving membrane resilience rather than merely suppressing the generation of reactive oxygen species [248,249].

Inhibition of ferroptotic signaling should be viewed as one component of broader efforts to preserve the structural integrity of the mitochondrial and endothelial membranes that sustain cellular homeostasis during reperfusion.

#### 4.1.5. Activating Adaptive Stress Responses

Among endogenous cytoprotective pathways, Nrf2 occupies a central position by coordinating transcriptional programs involved in antioxidant defense, mitochondrial function, xenobiotic metabolism, glutathione synthesis, and cellular repair [250,251]. Nrf2 activation enhances endogenous antioxidant and mitochondrial defense mechanisms before reperfusion [252,253]. Such interventions may be particularly attractive during ex vivo preservation, where controlled pharmacological activation can be achieved before implantation without exposing the recipient to prolonged systemic treatment.

#### 4.1.6. Restoring Endothelial Redox Competence

Preservation of endothelial function requires considerably more than maintaining vasodilation. Nitric oxide signaling coordinates vascular tone, mitochondrial respiration, platelet activation, leukocyte trafficking, and microvascular homeostasis, all of which become profoundly disturbed during transplantation [254,255].

Therapeutic strategies aimed at restoring nitric oxide bioavailability, preventing endothelial nitric oxide synthase uncoupling, preserving glycocalyx integrity, and limiting endothelial oxidative activation therefore address one of the central regulatory systems governing reperfusion biology. These interventions restore endothelial redox competence and microvascular homeostasis during reperfusion.

#### 4.1.7. Cell-Free Biological Therapies

The emergence of acellular therapeutic platforms represents another conceptual evolution in donor heart preservation. Rather than replacing injured cells, cell-free therapies harness endogenous biological mediators to modulate inflammation, preserve mitochondrial function, promote angiogenesis, and facilitate tissue repair while avoiding many of the logistical, manufacturing, and safety challenges associated with cellular transplantation [256,257].

Secretomes, conditioned media, recombinant protective proteins, and synthetic biological nanoparticles exemplify strategies to reshape the biological environment of the donor heart during preservation [258]. These therapies simultaneously target multiple redox-sensitive pathways.

#### 4.1.8. Extracellular Vesicles as Biological Reprogramming Platforms

Extracellular vesicles are among the most promising examples of multifunctional biological therapeutics [259]. Their molecular cargo—including proteins, lipids, microRNAs, metabolites, and mitochondrial regulatory molecules—can simultaneously modulate multiple aspects of cellular function, including redox metabolism, endothelial homeostasis, inflammatory signaling, mitochondrial quality control, and tissue repair [260,261].

Extracellular vesicles may become programmable biological platforms capable of coordinated molecular delivery during machine perfusion. The integration of vesicle-based therapies with machine perfusion may ultimately enable individualized biological reprogramming guided by dynamic redox phenotyping performed during ex vivo preservation.

These advances indicate that donor heart preservation is evolving from passive cytoprotection toward active biological optimization. Integration of redox engineering with dynamic phenotyping during machine perfusion may enable individualized therapeutic strategies tailored to the biological vulnerabilities of each donor heart [262].

### 4.2. Computational Reconstruction of Donor Heart Biology: From Molecular Signatures to Predictive Redox States

A major limitation of current donor evaluation is its reliance on reductionist measurements. Current donor evaluation relies predominantly on measurements that are intrinsically reductionist: ventricular function, hemodynamic stability, biochemical markers of myocardial injury, and increasingly, individual molecular biomarkers [260,263]. Each provides valuable information, yet each interrogates only a limited component of an exceptionally complex adaptive system. None directly measures the property that ultimately determines transplant success—the donor heart’s capacity to restore biological homeostasis after the profound perturbation imposed by ischemia, reperfusion, and implantation.

Recent advances in computational biology offer an opportunity to address this limitation. Rather than analyzing isolated variables, computational approaches seek to reconstruct the underlying biological state from which measurable molecular signatures arise. Artificial intelligence complements clinical judgment by integrating multidimensional biological information into mechanistically interpretable models [264]. The objective is no longer to identify better biomarkers of oxidative injury, but to estimate the adaptive redox state of the donor heart itself.

#### 4.2.1. Latent Biological States Beyond Individual Biomarkers

Many of the biological processes discussed throughout this review—including mitochondrial resilience, antioxidant reserve, endothelial competence, immunometabolic adaptation, and redox flexibility—cannot be measured directly. They are latent biological properties inferred from molecular and physiological measurements. Individual biomarkers, therefore, function as indirect observations rather than direct measurements of these underlying processes [265,266].

Identical concentrations of lipid peroxidation products, mitochondrial DNA, or inflammatory mediators may arise from markedly different biological contexts depending on the integrity of antioxidant systems, mitochondrial quality control, metabolic reserve, and endothelial adaptation. Conversely, donor hearts exhibiting similar clinical characteristics may possess profoundly different capacities for biological recovery.

Future donor assessment will therefore depend less on identifying singular biomarkers than on reconstructing these hidden biological states through simultaneous interpretation of multiple molecular layers. In this context, redox phenotyping should be regarded as the quantitative estimation of an integrated biological condition rather than the measurement of oxidative stress alone.

#### 4.2.2. Machine Learning as a Tool for Biological Pattern Recognition

Machine learning offers a fundamentally different analytical strategy from conventional statistical modeling. Traditional approaches evaluate predefined relationships between selected variables and clinical outcomes. Machine learning, in contrast, identifies complex multidimensional patterns that may not be apparent through human observation or hypothesis-driven analyses [267,268].

Applied to donor heart biology, machine learning can integrate metabolomic, lipidomic, proteomic, transcriptomic, extracellular vesicle, and physiological data into coordinated molecular signatures that reflect underlying biological organization [269]. Importantly, these algorithms need not identify individual biomarkers with independent predictive value. Instead, they recognize combinations of molecular changes that collectively define distinct biological phenotypes.

Such computational models may ultimately distinguish donor hearts with preserved adaptive capacity from those in which mitochondrial dysfunction, endothelial activation, inflammatory amplification, and oxidative imbalance have already converged toward irreversible biological deterioration. The principal contribution of machine learning, therefore, lies not in replacing established biomarkers but in revealing the higher-order biological relationships among them.

#### 4.2.3. Digital Twins as Dynamic Models of Donor Heart Biology

Among emerging computational technologies, digital twins represent perhaps the most ambitious approach to individualized donor assessment [270]. Rather than functioning as static predictive algorithms, digital twins are continuously updated computational representations that assimilate incoming biological information and modify their internal predictions accordingly [271].

Machine perfusion provides an ideal environment for such dynamic modeling. Sequential measurements of oxygen consumption, lactate metabolism, coronary vascular resistance, mitochondrial function, oxidative biomarkers, extracellular vesicles, cell-free DNA, and metabolomic profiles yield temporally resolved datasets that describe the evolving biological behavior of the graft [272,273]. Integration of these longitudinal data could generate dynamic models of graft recovery or deterioration and allow simulation of responses to alternative preservation or therapeutic strategies. However, digital twins in heart transplantation remain conceptual and will require prospective validation, standardized input variables, and demonstration of clinically meaningful incremental value before they can contribute to donor-selection decisions.

#### 4.2.4. Predictive Redox States and Biological Trajectories

Conventional predictive models estimate the probability of discrete clinical outcomes such as primary graft dysfunction, rejection, or mortality [274]. While clinically valuable, these endpoints occur relatively late in the biological continuum and provide limited guidance on the mechanisms underlying graft deterioration.

A redox-centered computational framework instead predicts biological trajectories before irreversible injury becomes established. Progressive restoration of mitochondrial respiration, normalization of metabolic flux, preservation of endothelial homeostasis, declining oxidative damage, and attenuation of inflammatory signaling collectively define a trajectory of biological recovery [275]. Conversely, persistent mitochondrial dysfunction, increasing lipid peroxidation, continued release of mitochondrial DNA, endothelial activation, and inflammatory amplification identify progression toward biological instability despite apparently preserved macroscopic function [276,277].

A particularly important question is whether the clinically relevant redox phenotype is defined by a static molecular state or by the graft’s capacity to recover during controlled perfusion. Serial measurements may therefore be more informative than isolated values. For example, declining oxidative-injury markers accompanied by normalization of metabolic indices and preservation of mitochondrial and endothelial function could represent a pattern of biological recovery, whereas persistent or worsening abnormalities despite adequate perfusion may indicate limited adaptive reserve. This dynamic-response concept should be tested prospectively rather than assumed from individual biomarker concentrations. Prediction shifts from estimating clinical events to modeling the evolution of the underlying biological system. Such trajectory-based analysis may identify opportunities for targeted intervention while adaptive capacity remains recoverable.

#### 4.2.5. Precision Donor Selection Based on Biological Phenotyping

The ultimate objective of computational redox phenotyping is not merely improved risk prediction but biologically informed decision-making. Current donor selection remains largely dependent on demographic characteristics, conventional clinical variables, and isolated indicators of organ injury. Although effective, this approach assumes that these variables adequately represent the graft’s intrinsic biological condition.

Future transplantation may instead rely on comprehensive biological characterization of each donor heart [261]. Multimodal molecular profiling integrated with physiological monitoring and computational modelling could generate individualized biological passports describing mitochondrial competence, antioxidant reserve, endothelial integrity, immunometabolic activation, and predicted responsiveness to therapeutic modulation during machine perfusion [278,279].

Such an approach would fundamentally redefine donor selection. Rather than categorizing organs as acceptable or unacceptable according to fixed criteria, transplantation strategies could be tailored to the specific biological vulnerabilities of each graft. Extended-criteria hearts exhibiting preserved adaptive redox capacity might undergo successful transplantation, whereas apparently favourable organs displaying computational signatures of irreversible biological instability could be identified before implantation. Precision donor selection would therefore evolve from static eligibility assessment toward dynamic biological stratification.

These developments suggest that the next major advance in transplantation will not arise from increasingly sophisticated algorithms alone, but from the convergence of computational biology, systems medicine, and redox science. Artificial intelligence will derive its greatest value not by predicting clinical outcomes, but by reconstructing the hidden biological architecture of donor hearts and transforming multidimensional molecular data into mechanistic understanding. Within the conceptual framework proposed in this review, computational redox phenotyping represents the final step in the evolution from descriptive donor assessment to biology-driven precision transplantation.

### 4.3. From Donor Selection to Biological Viability

Perhaps the most profound implication of contemporary redox biology is not the identification of new biomarkers or therapeutic targets, but the realization that the very concept of donor heart viability requires redefinition. Throughout the history of cardiac transplantation, viability has been inferred indirectly from demographic characteristics, structural integrity, hemodynamic performance, and selected biochemical measurements [228,280]. This approach has proved remarkably successful and has supported decades of excellent clinical outcomes. Nevertheless, it rests on a fundamental assumption: that clinical variables adequately represent the biological condition of the donor heart.

The concept of redox phenotyping does not replace conventional donor evaluation but extends it by incorporating biological information that remains largely inaccessible to traditional clinical assessment. This transition reflects a broader evolution from descriptive organ evaluation toward mechanism-based characterization of graft resilience. The principal differences between these complementary approaches are outlined in Table 5.

Hearts with comparable age, ventricular function, biomarker concentrations, and preservation times often demonstrate strikingly different responses to reperfusion, divergent susceptibility to primary graft dysfunction, and distinct long-term outcomes [298]. These observations indicate that the biological determinants of graft performance are only partially captured by conventional donor assessment. The variable that transplantation ultimately seeks to measure—the intrinsic capacity of the myocardium to recover after profound physiological stress—remains largely invisible to current clinical evaluation.

The conceptual framework proposed in this review suggests that this hidden property is fundamentally biological. It reflects the cumulative interaction of mitochondrial competence, antioxidant reserve, endothelial integrity, metabolic flexibility, immunometabolic adaptation, and the ability of the myocardium to re-establish redox homeostasis following ischemia and reperfusion [22,88]. Rather than representing independent determinants of donor quality, these processes converge to define a single systems-level property that may be described as biological viability.

The technologies discussed throughout this review naturally converge toward this objective. Redox biomarkers provide molecular evidence of oxidative adaptation; metabolomic and lipidomic profiling characterize ongoing metabolic organization; mitochondrial functional analyses reveal energetic competence; extracellular vesicles and cell-free nucleic acids describe intercellular communication and tissue instability; machine perfusion allows these biological processes to be observed dynamically rather than retrospectively; and computational modelling integrates these multidimensional datasets into coherent representations of organ physiology [299,300]. Individually, each technology contributes only a fragment of biological information. Collectively, they enable reconstruction of the adaptive state of the donor heart before implantation.

Importantly, the endpoint of this evolution is not increasingly sophisticated donor selection. The endpoint is the emergence of a fundamentally different decision-making framework. Instead of determining whether a donor satisfies predefined acceptance criteria, clinicians will increasingly evaluate whether the biological state of the organ is compatible with recovery, whether adaptive capacity can be enhanced during ex vivo preservation, and whether specific therapeutic interventions are likely to modify the predicted biological trajectory before implantation.

Within this paradigm, donor hearts cease to be classified as standard- or extended-criteria organs. Instead, they become biologically characterized systems with measurable adaptive potential. Chronological age may be outweighed by preserved mitochondrial resilience, prolonged ischemic time by intact endothelial competence, and elevated injury biomarkers by robust antioxidant reserve [301,302]. Conversely, organs fulfilling conventional acceptance criteria may exhibit computational signatures of exhausted biological adaptability despite apparently favourable clinical characteristics. Biological viability therefore becomes a dynamic property that can be quantified, monitored, and, increasingly, therapeutically modified.

Once viability is understood as a dynamic biological state rather than a fixed clinical characteristic, organ preservation also undergoes a conceptual transformation. Machine perfusion no longer functions solely as a preservation technology but becomes an experimental platform for biological interrogation and targeted intervention [303,304]. Therapeutic strategies evolve from passive protection against injury toward active restoration of mitochondrial function, endothelial homeostasis, and redox competence [305,306]. Donor assessment, organ preservation, and therapeutic optimization consequently become components of a single integrated biological continuum.

This emerging framework ultimately redefines the objective of precision transplantation. The goal is to complement conventional donor risk assessment with a more detailed characterization of the biological condition of each available donor heart, potentially improving estimation of its capacity for recovery and identifying opportunities for targeted optimization before implantation.

### 4.4. From Concept to Clinical Reality: Barriers to Implementing Precision Redox Transplantation

The conceptual framework presented throughout this review proposes a transition from conventional donor assessment toward biologically informed evaluation based on integrated redox phenotyping. Despite remarkable advances in molecular profiling, machine perfusion, and computational medicine, the transition from experimental proof-of-concept to routine clinical implementation remains a major obstacle [307,308]. The principal challenge is no longer the absence of candidate biomarkers or emerging technologies. Rather, it is the integration of these diverse sources of biological information into a robust, reproducible, and clinically actionable framework that supports transplantation decisions across healthcare systems.

Perhaps the greatest obstacle lies in the complexity of redox biology itself. Unlike conventional biochemical markers, redox homeostasis is inherently dynamic, compartmentalized, and context dependent. Oxidative modifications vary with metabolism, oxygen availability, inflammation, and therapeutic intervention, while antioxidant systems continuously modify the biological response. Consequently, individual measurements obtained at a single time point are unlikely to capture the adaptive state of the donor heart. Clinical implementation will require standardized multidimensional phenotypes that remain biologically meaningful across preservation strategies and clinical settings.

Translation of redox phenotyping from a conceptual framework to a clinically testable strategy requires definition of measurable domains, appropriate biological samples, sampling time points, and clinically relevant endpoints. Rather than searching for a single universal marker of graft quality, a more realistic approach is likely to combine complementary measures reflecting oxidative injury, antioxidant reserve, mitochondrial competence, metabolic recovery, and endothelial integrity. A proposed operational framework is summarized in Table 6.

Standardization represents the next critical step toward clinical implementation. Analytical variability currently exists at multiple levels, including sample acquisition, collection timing, tissue versus perfusate analysis, analytical platforms, normalization strategies, and data interpretation. These methodological differences substantially limit comparisons between studies and hinder independent validation of promising molecular signatures. International consensus regarding pre-analytical procedures, assay performance, quality control, and reporting standards will be essential before redox phenotyping can become a reliable component of donor evaluation.

Most available studies have focused on associations between individual biomarkers and short-term clinical outcomes, whereas far less attention has been devoted to determining whether integrated redox phenotypes represent biologically distinct states of the donor heart [60,309]. Establishing such phenotypes will require prospective studies combining molecular profiling with mechanistic investigations of mitochondrial function, endothelial adaptation, regulated cell death, and inflammatory responses. Validation should therefore extend beyond statistical prediction and demonstrate that computationally derived phenotypes correspond to reproducible biological mechanisms.

Achieving this objective will inevitably require large collaborative research networks. The biological heterogeneity of donor hearts, variations in procurement practices, differences in machine perfusion protocols, and relatively limited transplant volumes at individual institutions make adequately powered single-center studies unlikely to provide definitive answers [310]. International multicentre consortia integrating standardized biospecimen collection, harmonized molecular analyses, and uniform clinical outcome definitions will be indispensable for developing generalizable computational models and externally validated redox signatures.

Another major challenge concerns the integration of increasingly complex molecular datasets. Multi-omic platforms and functional physiological measurements describe complementary dimensions of donor biology. Their true biological value will emerge only when analyzed collectively rather than independently [311,312]. This transition will require computational frameworks capable of integrating heterogeneous datasets while preserving mechanistic interpretability. Future predictive models must therefore remain biologically transparent, allowing clinicians to understand not only the probability of graft dysfunction but also the molecular processes responsible for that prediction.

Comprehensive multi-omic profiling and advanced computational analyses inevitably increase the complexity and cost of donor assessment. Even modest improvements in donor utilization, reduction in primary graft dysfunction, optimization of machine perfusion strategies, or expansion of safely transplantable extended-criteria organs could substantially offset initial costs through improved graft survival and more efficient use of the limited donor pool. Formal health-economic analyses should therefore accompany future translational studies and evaluate the cost-effectiveness of biological phenotyping within the entire transplantation pathway rather than during donor assessment alone [313,314].

Implementation into clinical practice will also require an appropriate regulatory framework. As donor evaluation evolves from conventional laboratory testing toward integrated molecular diagnostics supported by artificial intelligence, questions regarding analytical validation, algorithm transparency, reproducibility, data governance, and clinical accountability become increasingly important. Future regulatory pathways should evaluate computational redox models not merely as software tools but as clinical decision-support systems whose recommendations directly influence organ allocation and therapeutic intervention.

Identifying these limitations is essential for defining realistic priorities for future research and clinical implementation. The major barriers and potential solutions are summarized in Table 7.

Even if redox phenotyping becomes analytically robust, its clinical implementation will ultimately depend on seamless integration into the time-critical transplantation workflow. Molecular analyses must generate results within clinically relevant decision windows, interfaces should provide interpretable outputs for multidisciplinary transplant teams, and computational recommendations must complement rather than delay existing allocation processes [326]. Consequently, practical feasibility will become as important as analytical performance.

Finally, the field must address a broader conceptual challenge. Redox phenotyping proposes a fundamentally different approach in which donor quality emerges from the interaction of dynamic biological networks rather than isolated clinical variables [233]. Adoption of this framework will therefore require not only technological innovation but also a shift in scientific thinking—from describing donor characteristics to quantifying biological adaptability.

**Figure 6:**
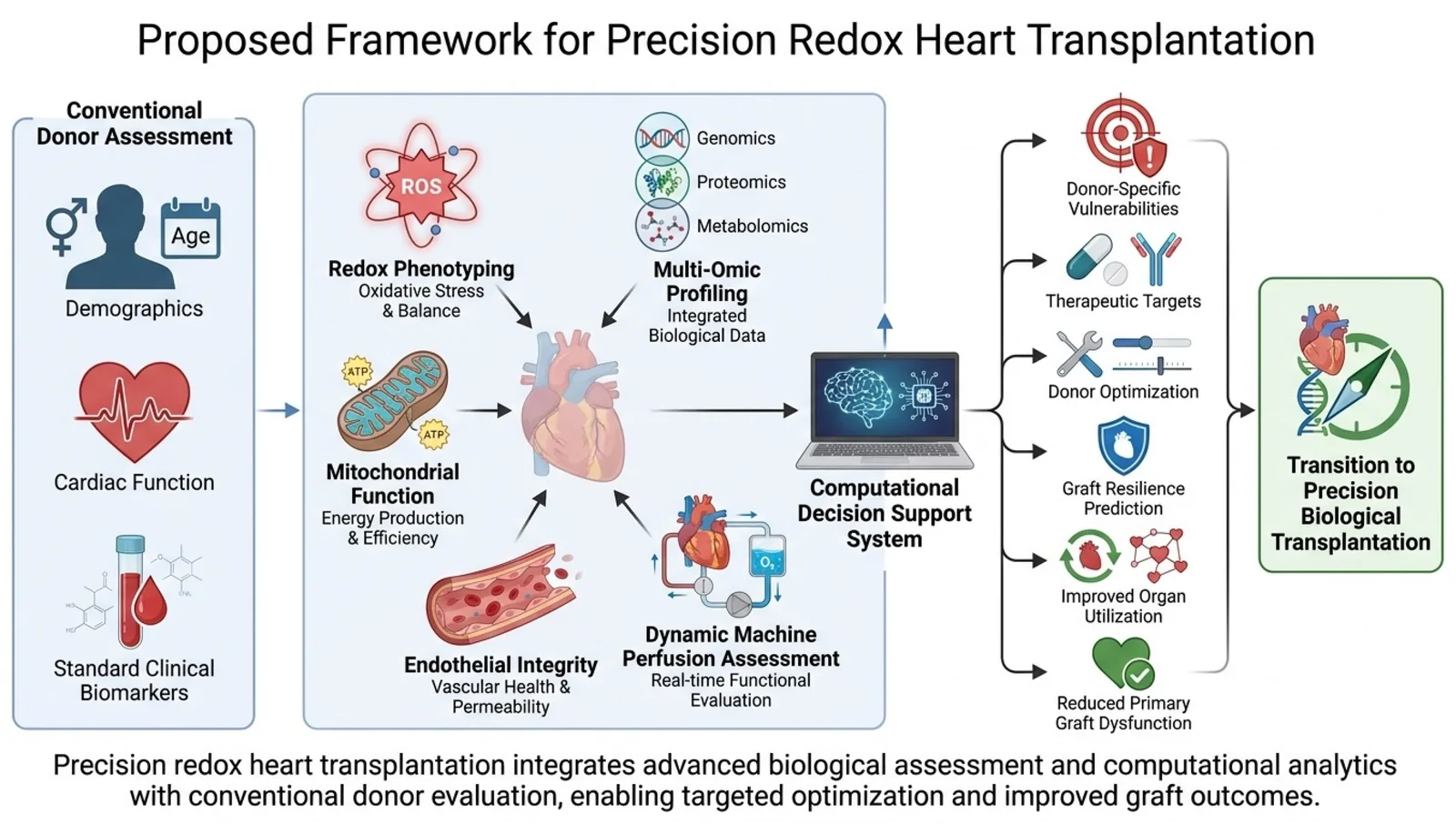
Precision Heart Transplantation. Figure legend: The figure illustrates the proposed framework for precision redox heart transplantation. Conventional donor assessment, based primarily on demographic characteristics, cardiac function, and standard clinical biomarkers, is expanded through integration of redox phenotyping, mitochondrial function, endothelial integrity, multi-omic profiling, and dynamic assessment during machine perfusion. These complementary data are incorporated into computational decision-support systems to identify donor-specific biological vulnerabilities and therapeutic targets before implantation. This strategy could support individualized donor optimization and more comprehensive assessment of graft resilience and provides a framework for investigating whether biologically informed donor assessment can improve organ utilization and reduce the risk of primary graft dysfunction.

The concepts discussed throughout this review converge into a proposed framework for precision donor-heart assessment in which conventional clinical evaluation is complemented by molecular phenotyping, dynamic assessment during machine perfusion, and computational integration. Figure 6 summarizes this transition from conventional donor assessment toward biologically informed precision heart transplantation.

### 4.5. Future Research Priorities for Clinical Validation of Redox Phenotyping

The transition from conceptual redox phenotyping to clinical application requires several questions to be addressed prospectively. First, which individual biomarkers or combinations of biomarkers most reproducibly characterize donor-heart redox state independently of conventional donor risk factors? Second, which biological compartment—donor blood, myocardial tissue, preservation solution, or machine-perfusion perfusate—provides the most clinically informative signal? Third, it remains unknown whether absolute biomarker concentrations or dynamic trajectories during machine perfusion better reflect graft resilience. Fourth, candidate redox phenotypes must be tested against clinically relevant outcomes, including primary graft dysfunction, requirement for mechanical circulatory support, early graft dysfunction, rejection, cardiac allograft vasculopathy, and survival. Fifth, the incremental predictive value of redox phenotyping should be established beyond conventional donor characteristics, echocardiography, hemodynamics, ischemic time, and currently available perfusion parameters. Finally, multicenter prospective studies will be required to determine analytical reproducibility, clinically meaningful thresholds, and whether phenotype-guided interventions improve organ utilization or recipient outcomes. Until these questions are addressed, redox phenotyping should be considered a hypothesis-generating and investigational framework rather than a validated tool for donor acceptance or rejection.

## 5. Conclusions

Further improvements in heart transplantation may depend not only on expansion of the donor pool but also on more precise characterization of donor-organ biology. Nevertheless, contemporary donor assessment remains largely based on clinical descriptors that indirectly estimate organ quality and provide only limited insight into the biological mechanisms that ultimately determine graft resilience after implantation.

Donor heart viability should be regarded as a dynamic biological state determined by interactions among mitochondrial function, endothelial integrity, metabolism, inflammation, and antioxidant capacity. Redox homeostasis provides the integrative framework linking these processes throughout donor management, preservation, and reperfusion.

Emerging molecular technologies and machine perfusion enable direct assessment of biological processes that are not captured by conventional clinical evaluation. Their integration provides the foundation for biologically informed donor assessment.

Redox phenotyping should not be interpreted as the introduction of another biomarker or diagnostic platform. Redox phenotyping assesses the donor heart’s capacity to maintain biological resilience rather than simply quantifying oxidative injury. In this emerging paradigm, the objective is not merely to detect oxidative injury but to determine whether the biological systems responsible for maintaining redox homeostasis remain sufficiently resilient to support functional recovery after transplantation. Such an approach recognizes that organs with similar clinical characteristics may possess fundamentally different capacities for adaptation, while hearts traditionally considered marginal may retain substantial biological reserve that is not captured by current evaluation strategies.

The integration of dynamic molecular profiling with machine perfusion and computational modeling further extends this concept beyond donor selection alone. Continuous assessment of mitochondrial performance, endothelial function, metabolic organization, and oxidative signaling creates opportunities not only to evaluate donor hearts more accurately but also to modify their biological state before implantation. Dynamic molecular assessment during machine perfusion may enable individualized therapeutic intervention before implantation.

Considerable scientific and translational challenges remain before this vision can be incorporated into routine clinical practice. Standardized molecular assays, prospective multicentre validation, integration of multi-omic datasets, interpretable computational models, and regulatory frameworks will all be required to establish biological phenotyping as a clinically robust decision-support strategy. Equally important will be demonstrating that biologically informed donor assessment improves organ utilization, reduces primary graft dysfunction, and expands transplantation without compromising recipient outcomes.

Heart transplantation is entering an era in which donor quality will increasingly be defined by biological function rather than static clinical descriptors. Redox phenotyping provides a conceptual framework for integrating oxidative biology, mitochondrial competence, endothelial integrity, and dynamic molecular assessment into precision donor evaluation. Whether this paradigm ultimately improves graft utilization and patient outcomes will depend on rigorous prospective validation, but it establishes a biologically grounded direction for the future of transplantation.

## Figures and Tables

**Table 1:** Biological Determinants of the Donor Heart Redox Phenotype Throughout Life.

Determinant	Principal Redox Mechanism	Biological Systems Affected	Contribution to Donor Heart Redox Phenotype	Representative Biomarkers	References
Aging	Mitochondrial dysfunction, impaired mitophagy	Mitochondria, endothelium	Reduced adaptive reserve	mtDNA, 8-OHdG	[26,33]
Hypertension	Ang II–NOX activation, eNOS uncoupling	Endothelium	Impaired vascular redox homeostasis	ADMA	[34,35]
Diabetes	AGE–RAGE signaling, PKC activation	Metabolism, mitochondria	Reduced metabolic flexibility	HbA1c, oxLDL	[36,37]
Obesity	Adipose inflammation, mitochondrial dysfunction	Immune–metabolic axis	Chronic low-grade oxidative inflammation	Leptin, adiponectin	[38,39]
Smoking	Exogenous ROS, oxidative DNA injury	Endothelium	Persistent oxidative injury	F2-isoprostanes	[40,41]
Air pollution	PM2.5/PM10/NOx-induced oxidative stress	Endothelium, innate immunity	Endothelial activation	MPO	[42]
Chronic inflammation	Cytokine–ROS amplification	Immune system	Persistent redox-inflammatory activation	IL-6, TNF-α	[43,44]

Abbreviations: 8-OHdG, 8-hydroxy-2′-deoxyguanosine; ADMA, asymmetric dimethylarginine; AGE, advanced glycation end products; Ang II, angiotensin II; eNOS, endothelial nitric oxide synthase; F2-isoprostanes, F2-isoprostanes; HbA1c, glycated hemoglobin; IL-6, interleukin-6; MPO, myeloperoxidase; mtDNA, mitochondrial DNA; NOx, nitrogen oxides; NADPH oxidase; oxLDL, oxidized low-density lipoprotein; PKC, protein kinase C; PM2.5, fine particulate matter with an aerodynamic diameter ≤ 2.5 μm; PM10, particulate matter with an aerodynamic diameter ≤ 10 μm, RAGE, receptor for advanced glycation end products; ROS, reactive oxygen species; TNF-α, tumor necrosis factor-α.

**Table 2:** Sequential Redox Events During Heart Transplantation.

Transplantation Phase	Dominant Biological Process	Principal Sources of Oxidative Stress	Major Redox-Dependent Consequences	Potential Therapeutic Strategies	References
Brain death	Neurohumoral activation and systemic inflammatory response	NADPH oxidases (NOX2/NOX4), dysfunctional mitochondria, uncoupled eNOS	Oxidative priming, mitochondrial dysfunction, endothelial activation, inflammatory signaling	Donor hemodynamic optimization, antioxidant strategies, hormonal replacement, mitochondrial protection	[92,93]
Organ procurement	Warm ischemia and interruption of oxygen supply	Impaired mitochondrial electron transport chain, xanthine oxidase	ATP depletion, ionic imbalance, initiation of ischemic injury	Rapid procurement, ischemic preconditioning, optimized cardioplegia	[73,94]
Cold preservation	Metabolic suppression and mitochondrial remodeling	Succinate accumulation, incomplete electron transport, impaired antioxidant defenses	Reduced metabolic reserve, mitochondrial vulnerability, oxidative predisposition	Optimized preservation solutions, hypothermic or normothermic machine perfusion, mitochondrial-targeted preservation	[74,95]
Reperfusion	Abrupt restoration of oxygen and mitochondrial reactivation	Reverse electron transport (Complex I), mitochondrial ROS burst, NOX activation	Lipid peroxidation, mPTP opening, calcium overload, endothelial dysfunction, regulated cell death	Mitochondrial-targeted antioxidants, mPTP inhibition, controlled reperfusion, modulation of inflammatory signaling	[96,97]

Abbreviations: ATP, adenosine triphosphate; eNOS, endothelial nitric oxide synthase; mPTP, mitochondrial permeability transition pore; NOX, NADPH oxidase; ROS, reactive oxygen species.

**Table 3:** Candidate Biomarkers for Clinical Redox Phenotyping of Donor Hearts.

Biological Domain	Representative Biomarkers	Biological Interpretation	Potential Clinical Application in Donor Assessment	Translational Readiness	Sampling Source	References
Oxidative stress	MDA, 8-OHdG, F2-isoprostanes	Lipid peroxidation and oxidative DNA damage	Baseline oxidative burden and susceptibility to ischemia–reperfusion injury	Clinical	Plasma	[172,173]
Direct ROS assessment	Electron paramagnetic resonance (EPR/ESR) spectroscopy with spin probes/traps	Direct detection and characterization of short-lived radical species and redox activity	Experimental/translational	Tissue/perfusate	Direct ROS assessment	[174,175]
Antioxidant capacity	GSH/GSSG ratio, total antioxidant capacity (TAC), SOD activity	Endogenous antioxidant reserve and redox buffering capacity	Identification of donor hearts with reduced resilience to oxidative stress	Emerging	Plasma	[176,177]
Thioredoxin system	Thioredoxin (Trx), thioredoxin reductase (TrxR) expression/activity, Trx redox state	Thiol-disulfide redox regulation and endogenous antioxidant reserve	Thiol-disulfide redox regulation and endogenous antioxidant reserve	Experimental/ translational	Tissue/ perfusate/ plasma	[178,179]
Mitochondrial injury	Cell-free mtDNA, cytochrome c, GDF-15	Mitochondrial dysfunction and impaired bioenergetics	Prediction of mitochondrial vulnerability and reperfusion injury	Emerging	Plasma	[180,181]
Endothelial integrity	Syndecan-1, heparan sulfate fragments, soluble thrombomodulin	Glycocalyx shedding and endothelial injury	Assessment of microvascular dysfunction and endothelial activation	Clinical/ Emerging	Plasma	[182,183]
Metabolic remodeling	Succinate, fumarate, lactate	Altered mitochondrial metabolism and ischemic adaptation	Evaluation of metabolic reserve and ischemic burden	Experimental	Perfusate/Plasma	[184,185]
Membrane lipid remodeling	Cardiolipins, oxidized phospholipids (OxPLs), oxLDL	Mitochondrial membrane integrity and lipid oxidation	Characterization of mitochondrial structural injury	Experimental	Tissue/Perfusate	[186,187]
Extracellular vesicles	Endothelial EVs, cardiomyocyte EVs, miR-21, miR-126, miR-499	Intercellular redox signaling and tissue injury	Integrated assessment of donor heart biological state	Experimental	Plasma/Perfusate	[188,189]

Abbreviations: 8-OHdG, 8-hydroxy-2′-deoxyguanosine; cfDNA, cell-free DNA; cf-mtDNA, cell-free mitochondrial DNA; EPR, electron paramagnetic resonance; ESR, electron spin resonance; EVs, extracellular vesicles; GSH, reduced glutathione; GSSG, oxidized glutathione; HNE, 4-hydroxynonenal; MDA, malondialdehyde; MPO, myeloperoxidase; mtDNA, mitochondrial DNA; oxLDL, oxidized low-density lipoprotein; ROS, reactive oxygen species; SOD, superoxide dismutase; TAC, total antioxidant capacity; Trx, thioredoxin; TrxR, thioredoxin reductase.

**Table 4:** Therapeutic Strategies for Redox Engineering of Donor Hearts.

Biological Target	Representative Therapeutic Strategy	Principal Mechanism of Action	Expected Biological Effects	Current Translational Readiness	Compatibility with Machine Perfusion	References
Mitochondrial oxidative stress	MitoQ, SkQ1, SS-31 (Elamipretide)	Selective mitochondrial ROS scavenging, preservation of cardiolipin, stabilization of electron transport chain	Reduced ROS generation, improved ATP synthesis, preservation of mitochondrial integrity	Preclinical; early clinical evaluation (SS-31)	High	[94,223]
Nrf2 signaling	Sulforaphane, Bardoxolone methyl, Dimethyl fumarate	Activation of Nrf2–ARE pathway and endogenous antioxidant gene transcription	Enhanced antioxidant defense, improved glutathione metabolism, reduced oxidative injury	Preclinical–early clinical	High	[224,225]
H_2_S/RSSH signaling	H_2_S donors; persulfide/hydropersulfide-generating strategies	Protein persulfidation, modulation of mitochondrial respiration and redox signaling, limitation of oxidative injury	Reduced I/R injury, preservation of mitochondrial and endothelial function, attenuation of oxidative and inflammatory stress	Preclinical/experimental	Potentially high	[226,227]
Nitroxyl (HNO) signaling	HNO donors (e.g., Angeli’s salt; experimental HNO-releasing compounds)	Redox-sensitive modification of protein thiols, modulation of myocardial Ca^2+^ handling and vascular signaling	Improved myocardial relaxation/contractile performance, vasodilation, and attenuation of I/R-associated injury	Preclinical/experimental	Potential; requires velidation	[228,229]
Succinate metabolism	SDH inhibition (malonate derivatives), controlled reperfusion strategies	Limitation of succinate oxidation and reverse electron transport	Reduced mitochondrial ROS burst during reperfusion	Experimental	High	[230]
Ferroptosis	Ferrostatin-1, Liproxstatin-1, Iron chelators	Inhibition of lipid peroxidation and iron-dependent oxidative injury	Preservation of membrane integrity and mitochondrial function	Experimental	High	[231,232]
Endothelial glycocalyx	Albumin, hydrocortisone, antithrombin, glycocalyx-stabilizing perfusates	Preservation of endothelial barrier function and mechanotransduction	Reduced endothelial activation, vascular permeability, and leukocyte adhesion	Emerging translational evidence	High	[141,233]
Nitric oxide signaling	NO donors, BH4 supplementation, soluble guanylate cyclase stimulators	Restoration of eNOS coupling and endothelial redox balance	Improved microvascular perfusion and endothelial function	Preclinical–clinical	High	[234,235]
Extracellular vesicle therapy	Mesenchymal stromal cell-derived extracellular vesicles	Delivery of regulatory miRNAs, proteins, and mitochondria-protective cargo	Immunomodulation, mitochondrial preservation, enhanced tissue repair	Emerging	High	[236]
Cell death pathways	Caspase inhibitors, RIPK1 inhibitors, inflammasome inhibitors	Modulation of apoptosis, necroptosis, and pyroptosis	Reduced cardiomyocyte death and inflammatory injury	Experimental	Moderate–High	[237]

Abbreviations: BH4, tetrahydrobiopterin; EVs, extracellular vesicles; HOPE, hypothermic oxygenated perfusion; HMP, hypothermic machine perfusion; HNO, nitroxyl; H_2_S, hydrogen sulfide; NMP, normothermic machine perfusion; NO, nitric oxide; NRF2, nuclear factor erythroid 2-related factor 2; ROS, reactive oxygen species, RSSH, hydropersulfides/reactive sulfur species containing a persulfide group.

**Table 5:** Paradigm Shift from Conventional Donor Assessment to Precision Redox Phenotyping.

Dimension	Conventional Transplant Paradigm	Precision Redox Phenotyping Paradigm	Clinical Impact	Dimension	References
Concept of donor quality	Anatomically and functionally acceptable organ	Biologically resilient organ with preserved adaptive capacity	Moves evaluation from morphology to biology	Concept of donor quality	[281,282]
Definition of donor age	Chronological age	Biological age reflected by cumulative oxidative, metabolic, inflammatory, and mitochondrial remodeling.	May improve characterization of potentially viable extended-criteria donors	Definition of donor age	[283,284]
Assessment of myocardial injury	Troponin release and echocardiography	Integrated redox injury signature incorporating oxidative damage, mitochondrial integrity, antioxidant reserve, and cell death pathways	May help distinguish potentially reversible biological stress from advanced injury	Assessment of myocardial injury	[285,286]
Assessment of cardiac function	Left ventricular ejection fraction and hemodynamics	Mitochondrial competence, metabolic flexibility, endothelial integrity, and microvascular function	May provide additional information regarding the capacity for functional recovery after reperfusion	Assessment of cardiac function	[287]
Biological information	Isolated clinical variables and single biomarkers	Multi-layer molecular profiling integrating redox biomarkers, metabolomics, proteomics, transcriptomics, extracellular vesicles, and cell-free DNA	Provides a more multidimensional representation of the biological state of the donor heart	Biological information	[288,289]
Timing of evaluation	Static assessment before procurement	Continuous biological monitoring during normothermic or hypothermic machine perfusion	Enables real-time assessment of organ recovery	Timing of evaluation	[290,291]
Decision methodology	Population-derived risk scores and clinical judgement	Mechanism-based prediction models integrating biological phenotypes and artificial intelligence	Supports individualized donor selection	Decision methodology	[288,292]
Role of machine perfusion	Organ preservation	Dynamic platform for biological assessment, targeted intervention, and continuous therapeutic modulation	Converts preservation into active organ optimization	Role of machine perfusion	[293,294]
Therapeutic strategy	Accept or decline the donor heart	Identify biological vulnerabilities and selectively target oxidative stress, mitochondrial dysfunction, endothelial injury, ferroptosis, and inflammation	Enables precision organ repair before transplantation	Therapeutic strategy	[295,296]
Ultimate objective	Maximize donor utilization	Maximize biological viability and long-term graft resilience	Transforms transplantation into a precision biological discipline	Ultimate objective	[297]

Abbreviations: AI, artificial intelligence; LVEF, left ventricular ejection fraction; ROS, reactive oxygen species.

**Table 6:** Proposed Operational Framework for Redox Phenotyping of the Donor Heart.

Domain	Candidate Measurements	Potential Sample/Source	What It May Indicate	Stage of Assessment	Current Limitation
Oxidative injury	8-OHdG, F2-isoprostanes, oxLDL, protein carbonyls	Blood, myocardium, perfusate	Accumulated oxidative injury	Donor/procurement/perfusion	Lack of transplant-specific thresholds
Antioxidant reserve	GSH/GSSG, GPX activity, thioredoxin system	Blood, tissue, perfusate	Capacity to restore redox homeostasis	Donor/perfusion	Assay standardization
Mitochondrial competence	mtDNA, respiratory capacity, membrane potential, ATP-related indices	Myocardium/perfusate	Energetic reserve and mitochondrial injury	Procurement/perfusion	Limited rapid clinical assays
Metabolic adaptation	Lactate kinetics, succinate, acylcarnitines, metabolomic profile	Perfusate/blood/tissue	Metabolic flexibility and recovery	Primarily machine perfusion	Dynamic thresholds undefined
Endothelial integrity	Syndecan-1, NO-related pathways, endothelial EVs, adhesion molecules	Blood/perfusate	Microvascular and endothelial injury	Donor/perfusion	Limited cardiac specificity
Inflammatory-redox signaling	IL-6, TNF-α, MPO, DAMPs	Blood/perfusate	Redox–inflammatory activation	Donor/reperfusion	Strong biological confounding
Integrated phenotype	Multimarker/multi-omic signature + functional data	Multimodal	Composite graft resilience	Across transplantation pathway	Requires prospective validation

Abbreviations: 8-OHdG, 8-hydroxy-2′-deoxyguanosine; ATP, adenosine triphosphate; DAMP, damage-associated molecular pattern; EVs, extracellular vesicles; GPX, glutathione peroxidase; GSH, reduced glutathione; GSSG, oxidized glutathione; IL-6, interleukin-6; MPO, myeloperoxidase; mtDNA, mitochondrial DNA; NO, nitric oxide; oxLDL, oxidized low-density lipoprotein; TNF-α, tumor necrosis factor-alpha.

**Table 7:** Translational Priorities for Implementing Precision Redox Phenotyping in Heart Transplantation.

Strategic Domain	Current Bottleneck	Scientific Gap	Priority Actions	Expected Clinical Impact	References
Redox biomarker qualification	Lack of standardized assays, reference ranges, and clinically validated thresholds	Limited reproducibility and poor comparability across transplant centers	International assay standardization, external validation, and establishment of clinically meaningful cut-off values	Reliable biomarker-guided donor assessment	[315,316]
Biological phenotyping	Reliance on isolated biomarkers that incompletely capture donor biology	Oxidative stress, mitochondrial dysfunction, endothelial injury, and immune activation remain fragmented into separate measurements	Development of integrated redox phenotypes combining oxidative, metabolic, inflammatory, and mitochondrial signatures	Comprehensive characterization of donor heart viability	[200,317]
Dynamic organ assessment	Predominantly static evaluation before implantation	Molecular responses evolve continuously during preservation and reperfusion	Continuous biomarker monitoring during normothermic and hypothermic machine perfusion	Real-time evaluation of organ recovery and therapeutic response	[191,318]
Multi-omics integration	Independent analysis of metabolomic, proteomic, transcriptomic, lipidomic, and extracellular vesicle datasets	Limited understanding of systems-level biological interactions	Systems biology approaches integrating multi-omics into unified biological networks	Mechanistic classification of donor hearts	[319]
Artificial intelligence	Small, heterogeneous, and poorly harmonized datasets	Limited ability to build robust predictive models for biological viability	International multimodal registries integrating clinical, imaging, perfusion, and multi-omics data	Precision prediction of graft function and long-term outcomes	[320,321]
Therapeutic targeting	Predominantly empirical antioxidant administration	Limited mechanistic targeting of mitochondrial dysfunction, ferroptosis, endothelial injury, and immunometabolic remodeling	Combination therapies tailored to the dominant biological phenotype identified during perfusion	Personalized biological optimization before implantation	[307,322]
Clinical validation	Scarcity of prospective biomarker-guided transplantation studies	Uncertain incremental value beyond conventional donor assessment	Multicenter randomized studies evaluating biomarker-guided donor selection and redox-targeted interventions	Evidence-based incorporation into transplantation guidelines	[315,323]
Clinical paradigm	Donor selection based primarily on clinical characteristics	Biological state is rarely incorporated into allocation decisions	Integration of precision redox phenotyping into donor evaluation, machine perfusion, and allocation algorithms	Transition from donor selection to precision biological optimization	[324,325]

Abbreviations: AI, artificial intelligence; RCTs, randomized controlled trials.

## Data Availability

No new data were created or analyzed in this study. Data sharing is not applicable to this article.

## Abbreviations

The following abbreviations are used in this manuscript:

8-OHdG	8-Hydroxy-2′-deoxyguanosine
ADMA	Asymmetric Dimethylarginine
AGE	Advanced Glycation End Products
AI	Artificial Intelligence
Ang II	Angiotensin II
ATP	Adenosine Triphosphate
BAX	BCL2-Associated X Protein
BAK	BCL2 Antagonist/Killer
BH4	Tetrahydrobiopterin
Ca^2+^	Calcium Ion
cfDNA	Cell-Free DNA
cf-mtDNA	Cell-Free Mitochondrial DNA
DAMPs	Damage-Associated Molecular Patterns
DNA	Deoxyribonucleic Acid
eNOS	Endothelial Nitric Oxide Synthase
EVs	Extracellular Vesicles
F2-Isoprostanes	F2-Isoprostanes
GSH	Reduced Glutathione
GSH/GSSG	Reduced-to-Oxidized Glutathione Ratio
HbA1c	Glycated Hemoglobin
HMGB1	High-Mobility Group Box 1
HMP	Hypothermic Machine Perfusion
HOPE	Hypothermic Oxygenated Machine Perfusion
IL	Interleukin
IL-1β	Interleukin-1 Beta
IL-6	Interleukin-6
IL-18	Interleukin-18
ISHLT	International Society for Heart and Lung Transplantation
LVEF	Left Ventricular Ejection Fraction
MDA	Malondialdehyde
MEDLINE	Medical Literature Analysis and Retrieval System Online
MeSH	Medical Subject Headings
MLKL	Mixed Lineage Kinase Domain-Like Protein
MPO	Myeloperoxidase
mPTP	Mitochondrial Permeability Transition Pore
mtDNA	Mitochondrial DNA
NADH	Nicotinamide Adenine Dinucleotide (Reduced Form)
NADPH	Nicotinamide Adenine Dinucleotide Phosphate (Reduced Form)
NCX	Sodium–Calcium Exchanger
NMP	Normothermic Machine Perfusion
NO	Nitric Oxide
NOX	NADPH Oxidase
NOx	Nitrogen Oxides
Nrf2	Nuclear Factor Erythroid 2-Related Factor 2
oxLDL	Oxidized Low-Density Lipoprotein
OXPHOS	Oxidative Phosphorylation
PKC	Protein Kinase C
PM2.5	Fine Particulate Matter with an Aerodynamic Diameter ≤2.5 μm
PM10	Particulate Matter with an Aerodynamic Diameter ≤10 μm
RAGE	Receptor for Advanced Glycation End Products
ROS	Reactive Oxygen Species
RyR2	Ryanodine Receptor 2
SERCA2a	Sarcoplasmic/Endoplasmic Reticulum Calcium ATPase 2a
SOD	Superoxide Dismutase
TAC	Total Antioxidant Capacity
TCA	Tricarboxylic Acid
TNF-α	Tumor Necrosis Factor-Alpha
